# A genomic atlas of gut clostridia: phylogeny, butyrate, and propionate production

**DOI:** 10.3389/fmicb.2026.1761627

**Published:** 2026-04-10

**Authors:** Laura Sola, Francesco Candeliere, Enrico Busi, Stefano Raimondi, Alberto Amaretti, Maddalena Rossi

**Affiliations:** 1 Department of Life Sciences, University of Modena and Reggio Emilia, Modena, Italy; 2 Biogest-Siteia, University of Modena and Reggio Emilia, Reggio Emilia, Italy

**Keywords:** butyrate, *Clostridia*, gut microbiome, metagenomics, propionate

## Abstract

**Introduction:**

*Clostridia* is a major microbial class in the human gut, crucial for fermenting undigested carbohydrates and proteins, which produce short-chain fatty acids essential for gut health and immune balance. This study revised the taxonomic classification and phylogeny of all the species of intestinal *Clostridia* catalogued in the Unified Human Gastrointestinal Genome database using a whole-genome approach and assessed butyrate and propionate producing species.

**Methods:**

A total of 1,897 *Clostridia* species, including those with recognised binomial nomenclature and those lacking formal taxonomic classification, were retrieved and reclassified using GTDB-Tk. Their phylogeny was determined by identifying, concatenating, and aligning the 120 ubiquitous single-copy proteins defined in the GTDB. Average amino acid identity (AAI), percentage of conserved proteins (POCP), and phylogenetic relationships were used to organize the species into genera and families. The presence of enzymes belonging to the biosynthetic pathways for butyrate and propionate production was investigated in all genomes with the tool GapSeq.

**Results:**

Reclassification of the genomes resulted in 404 recognised species and 1,493 species lacking formal taxonomic classification. *Oscillospirales* and *Lachnospirales* encompassed most of the species. The pathways leading to butyrate and propionate production were analyzed in their entirety, revealing 519 species as potential butyrate producers, 257 as potential propionate producers and 77 capable of producing both. To assess the abundance of each species, 151 faecal metagenomes of healthy subjects were profiled, indicating that butyrate producing *Clostridia* accounted on average for 28.0% of each microbiome.

**Conclusions:**

This study offers a comprehensive overview of intestinal *Clostridia* diversity, emphasising their role in gut ecosystems and their potential for butyrate and propionate production.

## 1 Introduction

*Clostridia* are a major class of bacteria within the phylum *Bacillota* that densely inhabits the human gut. They are known for their remarkable metabolic diversity, particularly in how they utilise substrates and employ fermentative pathways. The continuous identification of new anaerobic Gramme-positive bacteria within the class *Clostridia* and its subordinate levels has, over time, led to significant confusion in species classification and relationships.

Several major studies have attempted to resolve this issue by proposing taxonomic revisions, particularly within the genus *Clostridium*, through in-depth analyzes of shared genetic and phenotypic characteristics (Haas and Blanchard, 2020). The complexity and inconsistencies of *Clostridia* became especially evident with the advent of molecular techniques, which offered a more precise understanding of their evolutionary connexions. The key study by (Collins et al. 1994) presented a significant taxonomic revision of the genus *Clostridium* based on rRNA phylogenetic analysis. This work revealed that *Clostridium* was polyphyletic and proposed classifying the species into clusters that better reflect their evolutionary relationships. A later, thorough analysis of the faecal metagenome from 51 healthy individuals using MetaPhlAn3 identified 77 taxonomic units within the *Clostridia* class, each assigned a recognised binomial nomenclature, further underscoring the urgent need for taxonomic revisions to delineate genera more clearly (Candeliere et al., 2023). The growing interest in rectifying *Clostridia* taxonomy was also evident in several recent publications, ranging from the reclassification of the *Peptostreptococcaceae* (Bello et al., 2024) and specific *Clostridium* and *Butyrivibrio* species (Lawson et al., 2023; Fatahi-Bafghi, 2024) to the phylogenomic mapping of solventogenic *Clostridia* (Jensen et al., 2024). Interest in intestinal *Clostridia* stems primarily from their predominance in the gut and their crucial role in degrading and fermenting complex carbohydrates that escape digestion in the upper gastrointestinal tract. Once these carbohydrates reach the colon, their fermentation produces short-chain fatty acids (SCFAs) such as acetate, propionate, and butyrate. These SCFAs serve as a vital energy source for enterocytes and are crucial for maintaining gut function and immune homeostasis (Sivaprakasam et al., 2016).

Among the SCFAs, butyrate is especially beneficial for gut health. It is the primary energy source for intestinal epithelial cells and provides a first line of cellular defence against pathogens (Hodgkinson et al., 2023; Jacobson et al., 2018). Butyrate also strengthens the intestinal barrier, mitigates proinflammatory signalling, and modulates T-cell responses through epigenetic regulation via the inhibition of histone deacetylases (HDACs) (Lamas et al., 2019; Bach Knudsen et al., 2018). Recent studies also suggest that propionate's cellular mechanisms differ from those of butyrate, indicating specific metabolic roles (Kilner et al., 2012). Besides its function as an energy source, propionate provides cardiovascular protection by reducing hepatic lipid synthesis and blood cholesterol levels. It exerts several other beneficial physiological effects, including anti-lipogenic, cholesterol-lowering, anti-inflammatory, and anti-carcinogenic actions (Hosseini et al., 2011; Vinolo et al., 2011). Another important property is its ability to enhance satiety and modulate appetite through the activation of free fatty acid receptors FFA2 and FFA3, an effect of increasing interest given the global rise in obesity (Arora et al., 2011; Han et al., 2024).

**Figure 1:**
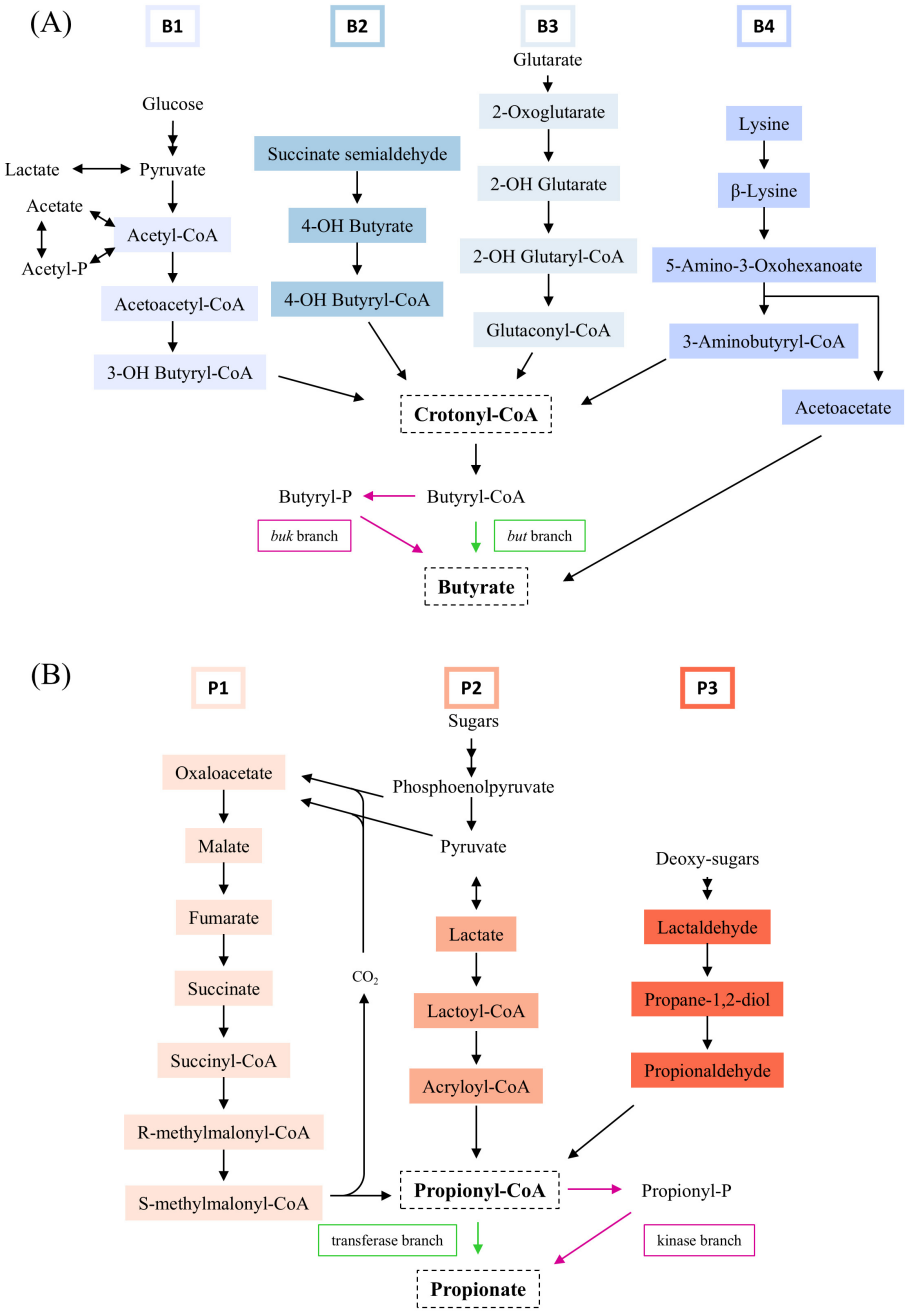
**Panel A**. Pathways for butyrate formation: B1, acetyl-CoA (thiolase); B2, succinate semialdehyde; B3, glutarate; B4, lysine. **Panel B**. Pathways for propionate formation: P1, succinate; P2, acrylate; P3, propanediol.

Butyrate can be produced by colonic bacteria either as the end product of carbohydrate fermentation, through the metabolism of lactate and acetate generated by primary fermenters, or as final product of few acetogens which use C1 compounds through the Wood-Ljungdahl pathway (Vital et al., 2014; Litty and Müller, 2021; Trischler et al., 2022). By utilising these fermentation products, butyrate-producing bacteria help prevent their accumulation, thus stabilising the gut environment. Four main pathways in the gut lead to butyrate production, starting from acetyl-CoA, glutarate, succinate semialdehyde, and lysine (Vital et al., 2014) (Figure 1A). The butyrate fermentation from acetyl-CoA is the most prevalent among gut microbes, especially *Clostridia*, involving the condensation of two acetyl-CoA molecules into acetoacetyl-CoA by thiolase and its subsequent reduction. Crucially, all four pathways converge with the reduction of crotonyl-CoA to butyryl-CoA. The final step, the conversion of butyryl-CoA into butyrate, is predominantly mediated by the enzyme butyryl-CoA:acetate-CoA transferase, encoded by the *but* gene. A less common reaction involves butyrate kinase, encoded by the *buk* gene (Vital et al., 2014; Louis et al., 2004).

Three main biochemical pathways contribute to propionate production: succinate, acrylate, and propanediol pathways (Reichardt et al., 2014) (Figure 1B). In the first pathway, succinate serves as the substrate for propionyl-CoA, being mainly sourced from the breakdown of sugars and proteins, though it is also a product of autotrophic growth in acetogens. (Reichardt et al., 2014; Shin et al., 2016; Fernández-Veledo and Vendrell, 2019). The acrylate pathway utilises lactate, converting it via lactoyl-CoA and acryloyl-CoA intermediates, with the key reaction catalysed by lactoyl-CoA dehydratase encoded by the *lcdA* gene. The propanediol pathway processes deoxy sugars into 1,2-propanediol, which is then metabolised to propionyl-CoA. All three pathways ultimately converge at the formation of propionyl-CoA, which is subsequently converted to propionate.

The cross-feeding of butyrogenic and propionigenic bacteria on SCFAs produced by other intestinal bacteria is also well documented (Louis and Flint, 2017; Duncan et al., 2004). Acetate utilisation can occur in bacteria via two distinct pathways: one involving acetate kinase and phosphate acetyltransferase, and another mediated by AMP-forming acetyl-CoA synthetase (Sun et al., 2020), whereas lactate utilisation involves D- and L-lactate dehydrogenases using NAD? or other electron acceptors, together with pyruvate-ferredoxin oxidoreductase (Duncan et al., 2004).

Proteins also serve as a significant carbon and energy source for some colonic *Clostridia*, which partake in the breakdown and fermentation of undigested proteins and peptides (Amaretti et al., 2019; Raimondi et al., 2021). This process primarily produces carbon dioxide, ammonia, and organic acids, with some amino acids also contributing to butyrate or propionate formation (Raimondi et al., 2021; Barker, 1981; Riedel et al., 2017). For instance, lysine fermentation by the commensal *Intestinimonas* and related taxa results in butyrate production (Bui et al., 2015, 2020). Similarly, *Clostridioides difficile* was reported to produce butyrate from glutamate-derived succinate semialdehyde, while alanine fermentation in *Clostridium propionicum* led to propionate production (Buckel, 2001; Gregory et al., 2021).

The present study characterised all the intestinal *Clostridia* catalogued in the Unified Human Gastrointestinal Genome database (UHGG; Almeida et al., 2021), which comprises 4,744 representative species from the human gut microbiome, to resolve taxonomic misclassifications and refine phylogenetic boundaries within this class. The manageable subset of intestinal *Clostridia* in UHGG facilitated a systematic genome-wide analysis combining phylogenomic and genome-based criteria, providing phylogenomic insight into hundreds of gut-associated *Clostridia* species. Given the significant role of intestinal *Clostridia* as SCFA producers, the main metabolic pathways related to butyrate and propionate production were investigated. The aim was to integrate existing knowledge of previously characterised butyrate-/propionate-producing species (Pryde et al., 2002; Louis and Flint, 2009; Louis et al., 2010; Louis and Flint, 2017; Magnúsdóttir et al., 2017; Zou et al., 2019) with poorly characterised and uncultivated ones, including metagenome-assembled genomes (MAGs). Finally, to estimate the potential impact of the clostridial community on intestinal butyrate and propionate levels, the abundances of *Clostridia* were assessed through the analysis of 151 faecal metagenomes from healthy subjects.

## 2 Materials and methods

### 2.1 Genomes retrieval

*Clostridia* genomes (*n* = 1,897) were retrieved from the UHGG catalogue v2.0.1 (http://ftp.ebi.ac.uk/pub/databases/metagenomics/mgnify_genomes/human-gut/v2.0.1/species_catalogue) and their taxonomic attribution was updated using GTDB-Tk v2.3.2 (classify_wf function) (Parks et al., 2022; Chaumeil et al., 2022) to align with GTDB taxonomy release R214. Representative genomes were subsequently obtained from GTDB (R214) when GTDB-Tk assignment allowed unambiguous matching (n = 1,706). For genomes with truncated or incomplete taxonomy (*n* = 191), UHGG v2.0.1 representatives were retained. GTDB genomes were prioritised due to their superior quality in terms of completeness and lower contamination levels. Among the 1,897 genomes, 404 were identified as representative species with established binomial nomenclature.

### 2.2 Phylogenomic analysis

To reconstruct the phylogeny of intestinal *Clostridia*, we identified, concatenated, and aligned the 120 ubiquitous single-copy proteins defined in the GTDB, using tools from GTDB-Tk v2.3.2 (Chaumeil et al., 2022). Phylogenetic trees were inferred using the PROTGAMMAAUTO model implemented in RAxML v8.2.12 (Stamatakis, 2014), rooted with *Bacillus licheniformis* ATCC 14580 (GCF_000011645.1), and visualised using iTOL v7.2.2 (Letunic and Bork, 2021). Bootstrapping was conducted with 1,000 replicates to assess tree robustness.

### 2.3 Genome-based relatedness indices

To assess genomic relationships among species with established nomenclature, the 404 representative species genomes were annotated using Prokka v1.14.5 (Seemann, 2014) and subjected to pairwise comparisons. The pairwise amino acid identity (AAI) was calculated using the EzAAI pipeline (Kim et al., 2021). The percentage of conserved proteins (POCP) was determined using the formula: [(C1 + C2)/(T1 + T2)] × 100, where C1 and C2 denote the number of conserved proteins shared between the two genomes being compared, and T1 and T2 represent the total number of proteins in the respective genomes (Qin et al., 2014). Conserved proteins were identified using Proteinortho v6.3.0 (Lechner et al., 2011), applying thresholds of 40% sequence identity and a minimum alignment length of 50%. Heatmaps were produced for AAI and POCP results, and it was assessed consistency with the current taxonomic attribution.

### 2.4 Screening for short-chain fatty acids producers

Butyrate and propionate biosynthetic pathways were screened across all 1,897 genomes using GapSeq v1.4.0 (Zimmermann et al., 2021) with default parameters. For butyrate, four pathways converging to crotonyl-CoA (acetyl-CoA, glutarate, lysine, and succinate semialdehyde) were evaluated, along with terminal steps via butyryl-CoA:acetate CoA-transferase or butyrate kinase. For propionate, three pathways (succinate, acrylate, and propanediol) leading to propionyl-CoA were screened, along with terminal CoA-transferase or kinase reactions.

### 2.5 Metagenomic samples

*Clostridia* species were analysed in a dataset of 151 metagenomes derived from faecal samples of healthy subjects from different geographic provenance (Supplementary Table 1). These metagenomes are publicly available in NCBI Sequence Read Archive (SRA), with the accession numbers PRJNA557323, PRJNA504891, PRJEB27308, PRJNA529124, PRJNA485056, PRJNA278393, PRJNA375935, PRJDB4176, PRJNA328899, PRJEB17784, PRJNA268964, and PRJEB7369 (Supplementary Table 1). The datasets were generated using whole-genome shotgun sequencing on Illumina paired-end platforms, yielding between 9.2 × 10^6^ and 1.0 × 10^10^ reads per sample, with read lengths ranging from 100 to 150 base pairs.

### 2.6 Composition analysis

The microbial composition of the 151 metagenomes was determined using the Kraken2 taxonomic sequence classifier (Wood et al., 2019), followed by Bracken analysis (Lu et al., 2017). We used a custom Kraken2 database based on the UHGG collection (Almeida et al., 2021) to achieve higher resolution in microbial profiling. Bracken was employed to refine Kraken2's initial classifications by re-estimating the number of reads assigned to each species in a sample. All analyses were conducted using the default parameters for k-mer length, minimizer length, and minimizer spacing.

## 3 Results

### 3.1 Phylogenomic resolution of intestinal *Clostridia*

The UHGG database encompassed genomes ascribed to 1,897 species of *Clostridia*, among which 246 with recognized binomial nomenclature (hereinafter indicated as recognized species, RS). Reclassification according to GTDB R214 returned 404 RS. The remaining 1,493 *Clostridia* genomes were not ascribed to any recognized species (no species, hereinafter indicated as NS) (Supplementary Spreadsheet 1). The representative genomes obtained following reclassification (*n* = 1,706 from GTDB R214; *n* = 191 from UHGG) exhibited high completeness, with 1,170 genomes (61.7%) showing >90% completeness and an additional 574 genomes (30.3%) exceeding 70% completeness. Notably, among the 404 RS genomes used for comparative analyses, 375 (92.8%) showed >90% completeness, with all remaining genomes exceeding 70%, ensuring high reliability for genome-based relatedness assessments and biochemical pathways reconstruction (Supplementary Spreadsheet 1).

**Figure 2:**
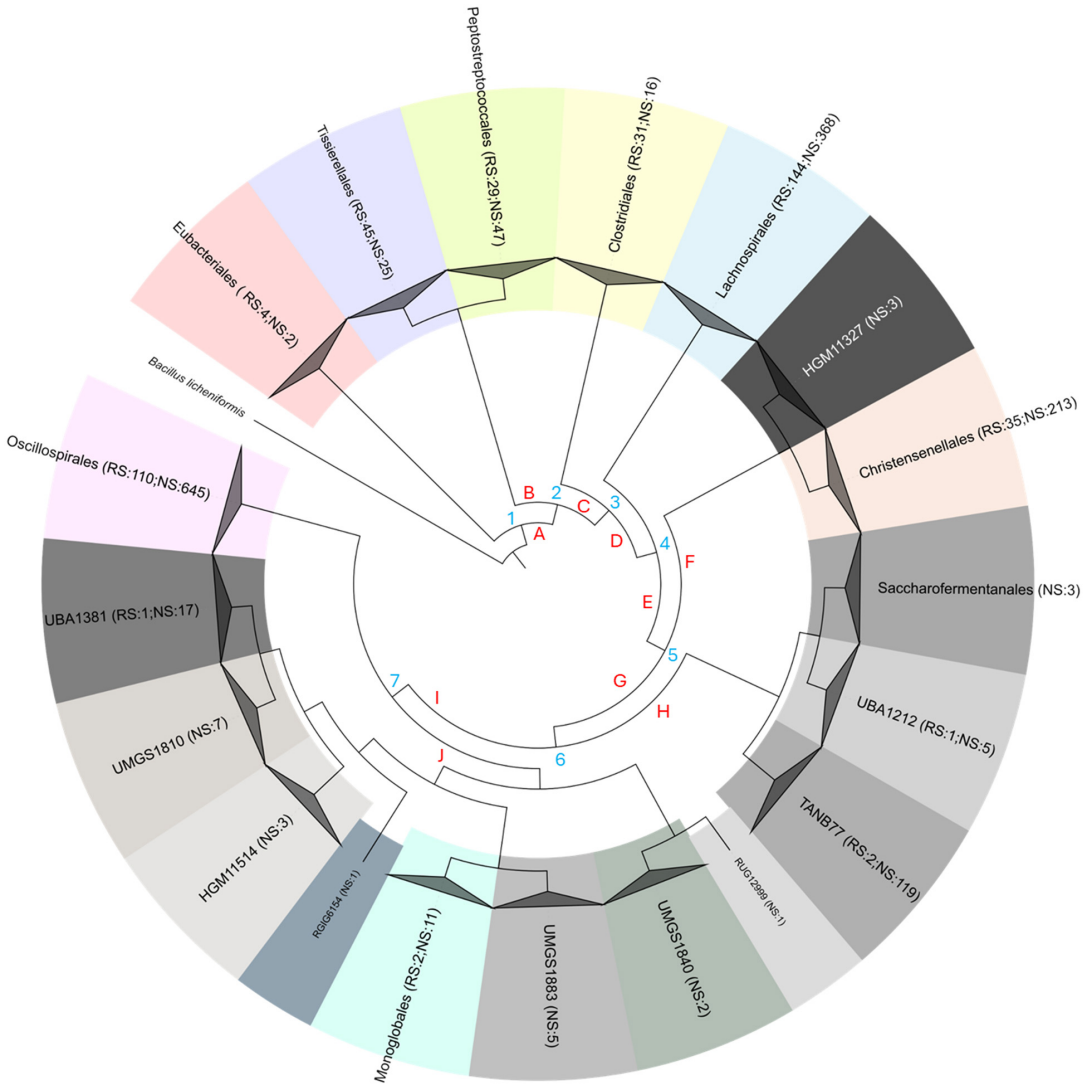
Phylogenetic tree of the 1,897 *Clostridia* retrieved from UHGG database, computed from the alignment of 120 ubiquitous single-copy proteins. Branch length is ignored, and leaves are collapsed to the order level for clarity of visualization. Lineages are in red, nodes in cyan. For each order, the number of NS and RS is given in brackets.

The 1,897 species of *Clostridia* belonged to 19 different orders. The alignment of 120 ubiquitous single-copy proteins yielded the phylogenetic tree reported in Supplementary Figure 1 and collapsed at the order level in Figure 2. *Eubacteriales* separated at node 1 from lineage A that gave rise to all the other 18 orders. In correspondence of node 2, lineage A split into lineage B that generated the orders *Peptostreptococcales* and *Tissierellales*, separated from a major one (C), which evolved in the other orders. Lineage C split at node 3 into *Clostridiales* and into branch D, which in turn split at node 4 into *Lachnospirales* from branch E. Taxa bipartition of branch E (node 5) led to branch F (orders HGM11327 and *Christensenellales*) and to branch G. In branch G, node 6 evolved to branch H (*Saccharofermentanales, UBA1212*, and *TANB77*) and to branch I. In branch I, node 7 gave rise to *Oscillospirales* and to branch J, which led to the orders *RUG12999, UMGS1840, UMGS1883, Monoglobales, RGIG6154, HGM11514, UMGS1810*, and *UBA1381*.

Most species of *Clostridia* belonged to the orders *Oscillospirales* (755) and *Lachnospirales* (512). Other species mainly belonged to *Christensenellales* (248), *Peptostreptococcales* (76), *Clostridiales* (47), *Monoglobales* (13), and *Eubacteriales* (6). Most of the species attributed to *Oscillospirales* were NS (85.4%, Supplementary Table 2), and consistently, NS were abundant within the main families *Acutalibacteraceae, Oscillospiraceae*, and *Ruminococcaceae* (85.9, 90.8, and 74.3%, respectively). The majority of *Lachnospirales* were ascribed to *Lachnospiraceae* (477/512) and represented by NS (71.9%). On the other hand, *Clostridiaceae* (*Clostridiales*), *Peptoniphilaceae* (*Tissierellales*), and *Peptostreptococcaceae* (*Peptostreptococcales*) mainly contained RS. Most of the species of *Christensenellales* were NS (85.9%). The richest family herein observed, *Borkfalkiaceae*, comprised 60 species with only 10 RS. The other main families (> 20 species; *CAG-74, UBA1242, CAG-138*, and *CAG-917*) commonly included NS. The order *TANB77* evolved in 3 families and mostly included NS (119/121). Its largest family, *CAG-508*, encompassed 109 species, including the 2 RS *Merdicola faecigallinarum* and *Scatovivens faecipullorum*. Eight orders (*UMGS1840, UMGS1883, HGM11514, RUG12999, UMGS1810, HGM11327, RGIG6154*, and *Saccharofermentanales*) did not encompass any RS (Figure 2, Supplementary Table 2).

### 3.2 Families and genera delineation

Taxonomic investigation focused on 404 representative species (RS). These RS were assigned to 11 GTDB orders and subjected to pairwise AAI comparisons to evaluate similarity within families and to delineate genera.

Among the 404 RS analyzed, the most represented order was *Lachnospirales* with 144 RS, followed by *Oscillospirales* with 110 RS; *Tissierellales* and *Christensenellales* included 45 and 35 RS, respectively, while *Clostridiales* and *Peptostreptococcales* comprised 31 and 29 RS. The remaining 8 RS were distributed across minor orders with fewer than 5 representatives. Overall, the 404 RS were assigned to 35 GTDB families, 8 of which contained more than 10 RS: *Lachnospiraceae* (138 RS, order *Lachnospirales*); *Ruminococcaceae, Acutalibacteraceae*, and *Oscillospiraceae* (44, 31, and 22 RS, respectively, order *Oscillospirales*); *Peptoniphilaceae* (42 RS, order *Tissierellales*); *Clostridiaceae* (31 RS, order *Clostridiales*); *Anaerovoracaceae* and *Peptostreptococcaceae* (14 RS each, order *Peptostreptococcales*). The lowest AAI value (minAAI) between RS was calculated for each family (Supplementary Spreadsheet 2). Among all intestinal clostridial families, *Peptoniphilaceae* exhibited the lowest minAAI (43.9%). Other families with more than 10 RS showed minAAI values up to 55.3%, as observed in *Peptostreptococcaceae*.

Genera were delineated considering the thresholds of 65% and 50% for AAI and POCP, respectively, according to GTDB genera nomenclature. The 404 RS belonged to 200 GTDB genera, 123 of which were singletons. Of the 77 genera with more than one species, 51 showed both minAAI and minPOCP values above the thresholds (Table 1, A), 12 met the minAAI threshold but not the minPOCP threshold (Table 1, B), 6 genera met the POCP threshold but not the AAI (Table 1, C), while 8 genera did not meet either threshold (Table 1, D).

**Table 1:** Delineation of nominal genera according to AAI and POCP thresholds.

Group	Taxa	minAAI	minPOCP
A	*Agathobacter, Alectryocaccomicrobium, Anaerobutyricum, Anaerostipes, Anaerotignum, Baileyella, Borkfalkia, Christensenella, Clostridioides, Clostridium E, Clostridium F, Clostridium G, Coprococcus, Coprococcus A, Diplocloster, Dorea A, Egerieicola, Eubacterium, Eubacterium I, Evtepia, Extibacter, Faecalibacterium, Fenollaria, Fimimonas, Finegoldia, Gallimonas, Gemmiger, Hungatella, Intestinimonas, Kallipyga, Lachnoanaerobaculum, Lachnoclostridium B, Lachnospira, Lentihominibacter, Murdochiella, Muricomes, Negativibacillus, Oliverpabstia, Paraclostridium, Parvimonas, Peptostreptococcus, Pseudoflavonifactor, Roseburia, Ruminococcus E, Scatavimonas, Scatomorpha, Sellimonas, Stomatobaculum, Terrisporobacter, Vescimonas, Zhenpiania*	YES	YES
B	*Acutalibacter, Agathobaculum, Anaerotruncus, Bariatricus, Blautia, Clostridium Q, Dysosmobacter, Eisenbergiella, Faecalimonas, Fournierella, Limiplasma, Mediterraneibacter*	YES	NO
C	*Anaerococcus, Butyribacter, Eubacterium R, Ezakiella, Massilioclostridium, Peptoniphilus A*	NO	YES
D	*Blautia A, Clostridium, Clostridium J, Enterocloster, Lachnoclostridium A, Marvinbryantia, Ruthenibacterium, Sarcina*	NO	NO

In some genera (e.g. *Anaerotruncus, Dysosmobacter, Butyribacter*, etc.), the under-threshold values were attributable to a single species showing lower similarity with one or more members of its genus (Supplementary Table 3, Supplementary Spreadsheet 2). In other genera (e.g. *Blautia, Blautia A, Clostridium, Clostridium J, Eisenbergiella, Enterocloster, Ezakiella, Lachnoclostridium A, Peptoniphilus A, Sarcina*, etc.) the exclusion of certain species and/or the subdivision of the nominal genus into two or three distinct groups would result in clusters that meet the established thresholds for genus delineation (Supplementary Table 3). Additionally, the merging of some species belonging to different nominal genera could give rise to 4 putative genera exceeding both thresholds (Supplementary Table 3).

### 3.3 Profiling of clostridial community in gut microbiomes

The abundance and prevalence of the 1,897 intestinal *Clostridia* species within the human gut microbiota was evaluated through the analysis of 151 faecal samples from healthy subjects (Supplementary Table 1). The profiling carried out with Kraken2 and Bracken identified 4,602 GTDB bacterial species, including all the 1,897 species of *Clostridia*, each of them being identified in no fewer than 10 samples. Of the 404 RS, 119 were detected in all 151 metagenomes. The abundance of *Clostridia* ranged between 9.7 and 88.1% of total bacteria, with a mean of 44.3%. On average, the 1,493 NS accounted for 56.1% of the total relative abundance of *Clostridia*.

**Figure 3:**
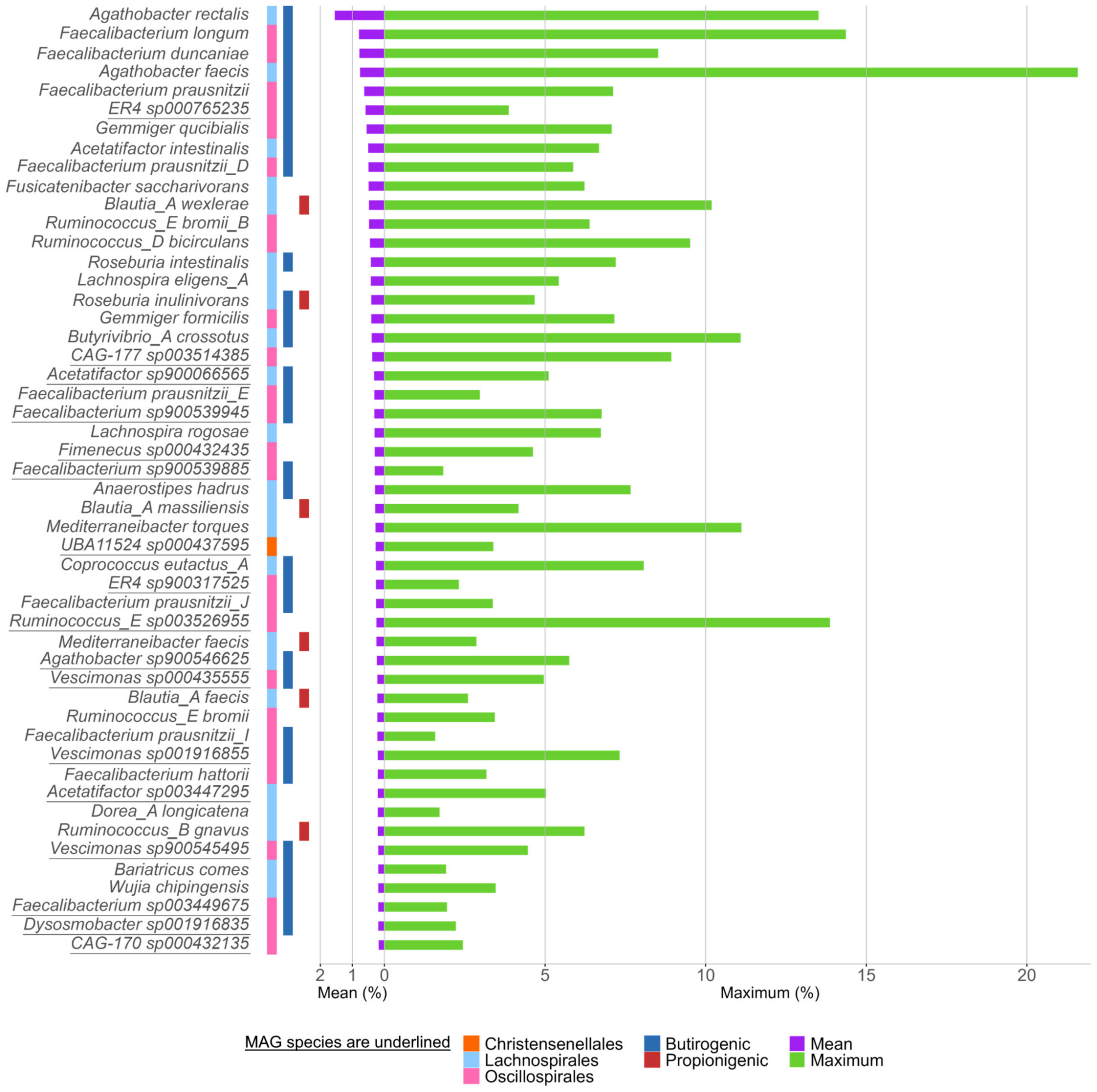
Relative abundance of the top 50 bacterial species, grouped by taxonomic order. Bars show mean (purple) and maximum (green) relative abundances. Colored tiles indicate the bacterial order and metabolic potential for butyrate and propionate production. Underlined names indicate MAGs.

The orders *Oscillospirales* and *Lachnospirales*, which encompassed most *Clostridia* species, were quantitatively the most abundant (on average 22.6 and 18.3%, respectively). The 50 most abundant species of *Clostridia* generally belonged to *Lachnospirales* (26) and *Oscillospirales* (23) and were mostly RS (33) (Figure 3). *Agathobacter rectalis, Faecalibacterium longum, Faecalibacterium duncaniae*, and *Agathobacter faecis* accounted on average for 1.55, 0.80, 0.78, and 0.76%, respectively, and reached remarkably high levels in single samples (13.5, 14.4, 8.5, and 21.6%, respectively) (Supplementary Spreadsheet 1). Other frequently occurring species that reached high abundance in individual samples were *Ruminococcus E sp003526955* (max 13.9%), *Mediterraneibacter torques* (max 11.1%), *Butyrivibrio A crossotus* (max 11.1%), and *Blautia A wexlerae* (max 10.2%). Other dominant species, with a mean abundance ranging between 0.4 and 0.7%, were *Faecalibacterium prausnitzii, ER4 sp000765235* (the most abundant NS), *Gemmiger qucibialis, Acetatifactor intestinalis, Faecalibacterium prausnitzii D, and Fusicatenibacter saccharivorans*.

### 3.4 Reconstruction of butyrate pathways

#### 3.4.1 Key reactions from crotonyl-CoA to butyrate: the but and buk branches

We reconstructed the metabolic pathways for butyrate production across the *Clostridia* class by screening for genes encoding butyryl-CoA dehydrogenase (which converts crotonyl-CoA to butyryl-CoA) and the downstream enzymes that complete butyrate synthesis. Two alternative routes exist: the butyryl-CoA:acetate-CoA transferase pathway (*but* branch) and the phosphate butyryltransferase/butyrate kinase pathway (*buk* branch). Among 771 genomes encoding butyryl-CoA dehydrogenase, 687 genomes (234 RS) possessed complete pathways to butyrate: 605 species encoded the *but* branch, 268 the *buk* branch, and 187 encoded both pathways. However, the distribution of these pathways varied widely across orders of *Clostridia* (Supplementary Spreadsheet 3).

Nearly all *Clostridiales* possessed both branches, whereas the vast majority of *Christensenellales* lacked them entirely, except for the species of *Christensenella, Avichristensenella, Scybalosoma*, and a few *Limiplasma*, which encoded the *but* branch.

Approximately half of *Lachnospirales* species harbored *but* or *buk* branches. The *but* branch was generally complete in most or all species of *Anaerotignum, Agathobacter, Alectryocaccobium, Anaerobutyricum, Anaerostipes, Anaerocolumna, Butyribacter, Clostridium Q, Coprococcus A, Eubacterium F, Copromonas, Frisingicoccus, Lachnoanaerobaculum, Mediterraneibacter A, Metalachnospira, Murimonas, Pararoseburia, Roseburia, Pseudoroseburia, Scybalocola, Dorea A*, and in some species of *Blautia, Lacrimispora, Oribacterium*, and *Ventrisoma*. The *buk* branch characterized all or most species of *Acetatifactor, Butyrivibrio, Eubacterium G, Wujia*, and some *Extibacter*. Both branches were present in species of *Coprococcus, Eisenbergiella, Eubacterium I, Hungatella, Scatomonas*, and various other genera. The genus *Enterocloster* was remarkably heterogeneous, comprising NS encoding *but, buk*, or both.

Within *Oscillospirales, but* and *buk* branches were less common but still widespread among known butyrate producers. The *but* branch characterized most species of *Agathobaculum, Butyricicoccus, Dysosmobacter, Evtepia, Intestinimonas, Onthomonas, Pseudoflavonifractor, Scatomorpha, Pseudoscilispira, Faecalibacterium, Pygmaiobacter*, and some *Anaerotruncus, Angelikisella*, and *Limivicinus*. The *buk* branch was present in most *Gemmiger* and in some *Fournierella, Pseudobutyricicoccus, Anaerotruncus*, and *Angelikisella*. Both branches were detected in most *Vescimonas, Lawsonibacter, Flavonifractor, Ventrusia*, and in some *Enterenecus, Dysosmobacter*.

In *Peptostreptococcales*, the majority of species possessed *but* and/or *buk*, except for a few genera (*Romboutsia, Peptacetobacter, Filifactor*) and a few NS. Most members of *Clostridioides, Paraclostridium, Peptostreptococcus*, and related taxa encoded both branches. The *but* branch was complete in *Eubacterium T, Fimisoma, Lentihominibacter*, and *Criibacterium*, while *Intestinibacter* encoded only the *buk* branch.

*Saccharofermentanales*, a small order with only three NS, included two species predicted to harbor both *but* and *buk*.

Most *Tissierellales* were predicted to produce butyrate, with the main exceptions being *Ezakiella, Finegoldia, Fenollaria, Neofamilia*, and *Parvimonas*. The *but* branch was found to be complete in *Anaerotruncus, Anaerococcus, Kallipiga, Murdochiella, Peptoniphilus A, Peptoniphilus B, Peptoniphilus C*, and *Peptoniphilus E*. Both branches were detected in *Tissierella, Sporanaerobacter, Anaerosalibacter, Sedimentibacter*, and several other NS.

Neither branch was detected in *Monoglobales* or in other unclassified clostridial lineages (e.g., TANB77, UBA121).

Notably, several species encoded *but* or *buk* genes but lacked butyryl-CoA dehydrogenase or phosphate butyryltransferase. Specifically, 193 species possessed *but* without butyryl-CoA dehydrogenase, mainly among *Christensenellales, Lachnospirales*, and *Oscillospirales*, while 125 species encoded *buk* and lacked one or both of these upstream enzymes.

#### 3.4.2 Routes yielding crotonyl-CoA: acetyl-CoA, lysine, succinate semialdehyde, and glutarate pathways

**Figure 4:**
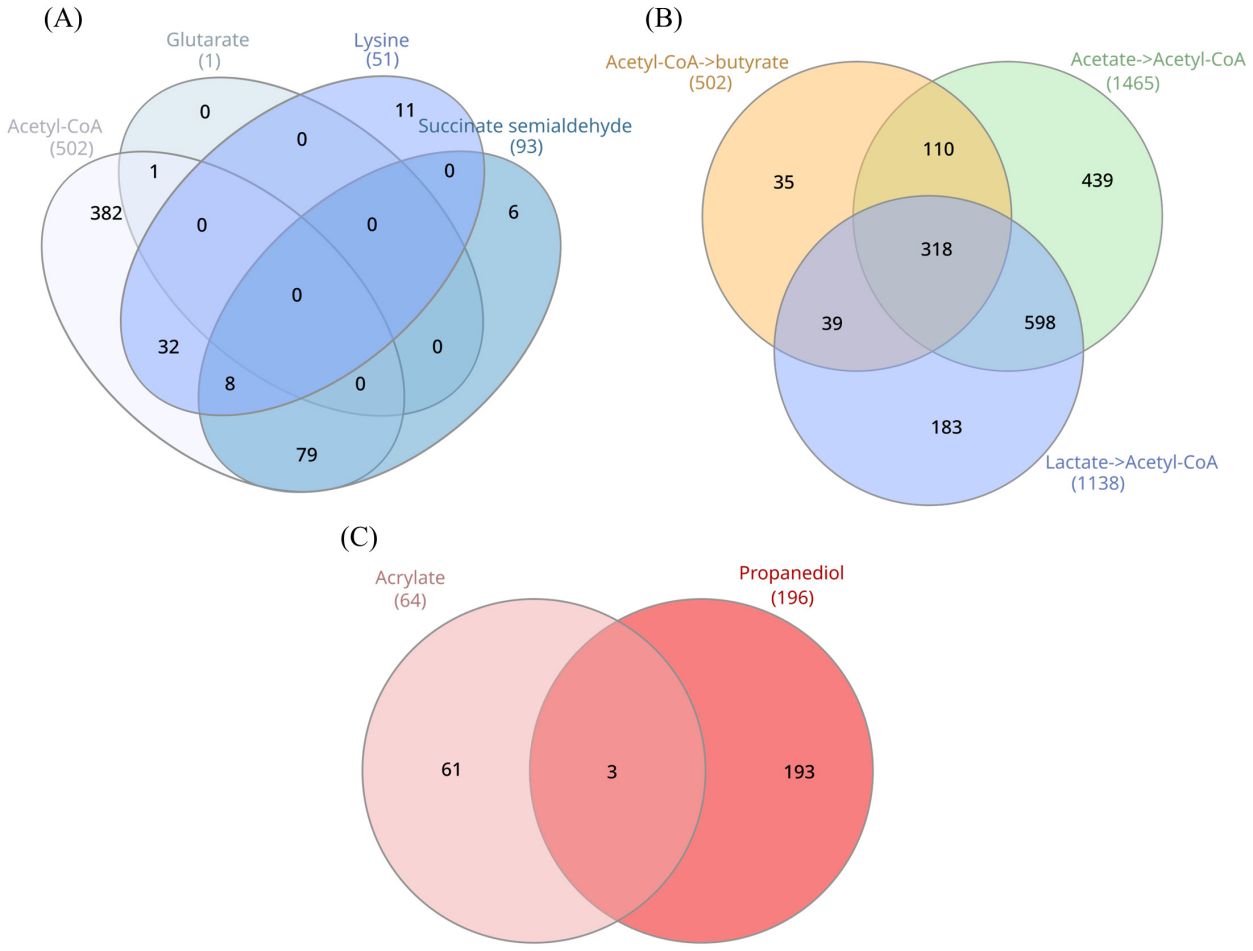
Distribution of predicted complete metabolic pathways in clostridial species (RS + NS). Pathways leading to crotonyl-CoA and then to butyrate **(A)**. Acetyl-CoA to butyrate, acetate to acetyl-CoA, and lactate to acetyl-CoA pathways **(B)**. Propionyl-CoA to propionate pathways **(C)**.

The main metabolic routes yielding crotonyl-CoA, i.e. acetyl-CoA (thiolase), lysine, succinate semialdehyde, and glutarate pathways, were reconstructed in the clostridial genomes. For 519 of the 687 species harboring complete *but* or *buk* branches, at least one complete pathway yielding crotonyl-CoA was identified (Figure 4). The number increased to 569 including those species lacking one metabolic block (Supplementary Figure 2A).

**Figure 5:**
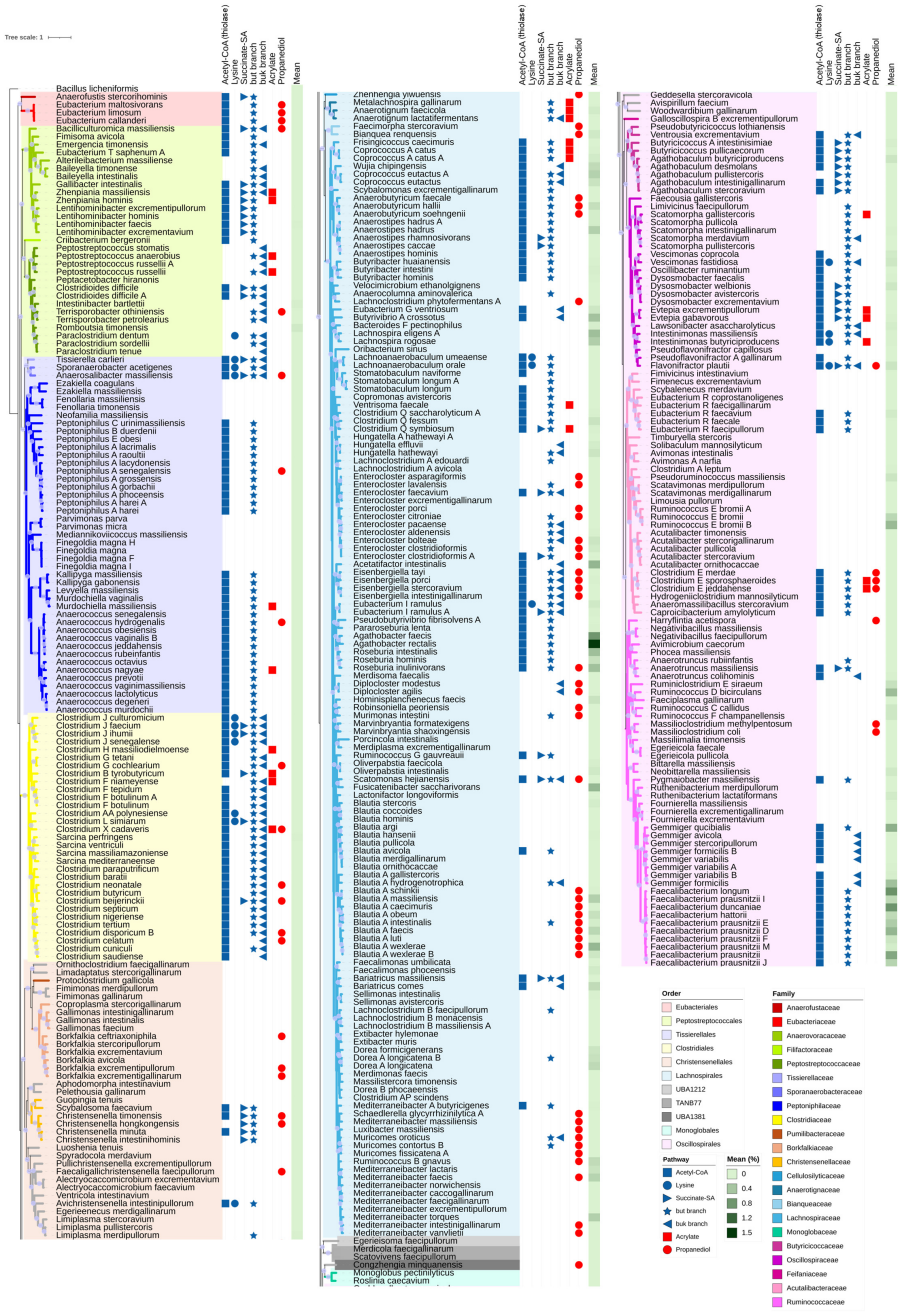
Phylogenetic distribution of butyrate and propionate biosynthetic pathways in RS *Clostridi*a species rooted on *Bacillus licheniformis*. Label backgrounds are colored by taxonomic order, while branch colors represent family-level classification (unassigned families shown in gray). Gray dots on branches indicate bootstrap support values >90. Symbols adjacent to species names indicate the presence of complete biosynthetic pathways: blue symbols denote butyrate production pathways (squares: acetyl-CoA pathway; circles: lysine pathway; triangles: succinate semialdehyde pathway), while red symbols indicate propionate production pathways (squares: acrylate pathway; circles: propanediol pathway). The glutarate pathway for butyrate and succinate pathway for propionate were not detected in any species and are therefore not represented.

The metabolic route transforming acetyl-CoA into crotonyl-CoA was complete in 521 species, of which 188 were RS (Figure 5). The presence of the genes participating in the transformation of acetyl-CoA to crotonyl-CoA was associated with the presence of complete *buk* and/or *but* branches to yield butyrate in 502 species (Figure 4A). Conversely, 185 species, widespread across all the orders, carrying either the *buk* or *but* branch, showed an incomplete acetyl-CoA–to–crotonyl-CoA pathway, lacking at least one metabolic block.

The pathway transforming lysine into crotonyl-CoA, including *ato*, was complete in 51 species (Figure 4A). The presence of this pathway was always accompanied by a complete *but* branch and in many cases also by the *buk* branch. In 11 species (*Paraclostridium dentum* being the only RS), lysine–to–butyrate conversion was accompanied by interruptions in the acetyl-CoA–to–crotonyl-CoA route.

The succinate semialdehyde pathway was complete in 93 species (Figure 4). RS harboring the complete pathway were found in *Christensenellales* (*Christensenella, Scybalosoma*), *Clostridiales* (*Clostridium, Clostridium B, Clostridium J, Clostridium L*), *Eubacteriales* (*Anaerofustis*), *Lachnospirales* (*Anaerostipes, Bariatricus, Clostridium Q, Enterocloster, Ruminococcus G, Scatomonas*), *Oscillospirales* (*Agathobaculum, Butyricicoccus, Butyricicoccus_A, Dysosmobacter, Evtepia, Flavonifractor, Anaerotruncus*), *Peptostreptococcales* (*Bacilliculturomica, Gallibacter, Lentihominibacter, Zhenpiania, Clostridioides*), and *Tissierellales* (*Anaerosalibacter, Tissierella*) (Figure 5). Except for rare cases, the succinate semialdehyde pathway co-occurred with a complete *but* branch and, less frequently, *buk*. Most species also encoded acetyl-CoA–to–crotonyl-CoA pathway (Supplementary Spreadsheet 3).

The glutarate pathway was complete only in one NS of *Saccharofermentanales*, while 76 species displayed it with one block missing, usually 2-oxoglutarate reductase (EC 1.1.1.399). These species mainly belonged to *Oscillospirales* (e.g. *Lawsonibacter, Oscillibacter, Pygmaiobacter, Clostridium E*), *Lachnospirales* (e.g. *Clostridium Q, Copromonas, Stomatobaculum, Ventrisoma*), and *Tissierellales* (e.g. *Anaerococcus, Kallipyga, Murdochiella, Peptoniphilus A*). With few exceptions, the glutarate pathway co-occurred with a complete *but* branch, in some cases *buk*, and a full acetyl-CoA–to–crotonyl-CoA route.

The potential utilization of acetate and lactate for butyrate production was also investigated, as both routes converge into the thiolase pathway. Enzymes involved in acetate and lactate metabolism were identified in numerous species (1,465 and 1,138, respectively) (Figure 4B), showing substantial overlap with each other and with those predicted to convert acetyl-CoA into butyrate. Of 502 species predicted to convert acetyl-CoA into butyrate, 428 had the genes for acetate metabolism and 357 had those for lactate metabolism, with 318 presenting both routes (Figure 4B).

### 3.5 Reconstruction of propionate pathways

The metabolic routes yielding propionate were inferred. The ability to convert propionyl-CoA to propionate through two possible routes was predicted in 1,469 species (361 RS). The route involving phosphate propionyltransferase followed by propionate kinase was complete in 1,423 species, the one involving propionyl-CoA:lactate/acetate transferase was found in 255 species, with 209 species encoding both branches (Supplementary Spreadsheet 3).

The main metabolic routes yielding propionyl-CoA, i.e. succinate, acrylate, and propanediol pathways, were reconstructed. Of the 1,469 species predicted to get propionate from propionyl-CoA, a complete pathway was predicted only in 257 species. The number increased to 659 including those species lacking one metabolic block (Supplementary Figure 2B). Although methylmalonyl-CoA carboxytransferase was widespread across the class *Clostridia* (predicted in 1,458 species), the succinate pathway leading to propionyl-CoA was consistently interrupted at the level of at least three metabolic blocks.

The acrylate pathway leading to propionyl-CoA was complete in 64 species (24 RS), but this number increased to 315 when including those lacking one metabolic block. The missing steps most frequently involved propionate:lactoyl-CoA transferase (115 species) or lactoyl-CoA dehydratase (134 species). The 64 species with a complete pathway were distributed across the orders *Clostridiales* (e.g. *Clostridium, Clostridium_F, Clostridium_H*, and *Clostridium_X, Eubacteriales* (*Garciella*), *Lachnospirales* (e.g. *Anaerotignum, Metalachnospira, Clostridium Q, Coprococcus A, Frisingicoccus, Ventrisoma*), *Oscillospirales* (e.g. *Clostridium E, Agathobaculum, Evtepia, Intestinimonas, Lawsonibacter, Scatomorpha, Vescimonas*), *Peptostreptococcales* (e.g. *Lentihominibacter, Zhenpiania, Peptostreptococcus*), and *Tissierellales* (*Anaerococcus, Murdochiella, Peptoniphilus, Sedimentibacter*). All these 64 species were predicted to produce propionate from propionyl-CoA. Notably, 57 out of the 64 species also presented at least one lactate dehydrogenase, enabling the connection between glycolysis and propionate production.

The propanediol pathway leading to propionyl-CoA was complete in 196 species (71 RS), but this number rose to 482 when including those lacking one block, most commonly propanal dehydrogenase (Figure 4C, Supplementary Figure 2B). Species harboring the complete pathway were distributed across *Christensenellales* (e.g. *Borkfalkia, Faecaligallichristensenella, Christensenella*), *Clostridiales* (e.g. *Clostridium, Clostridium G, Clostridium X*), *Eubacteriales* (*Eubacterium*), *Lachnospirales* (e.g. *Bianquea, Fecimorpha, Zhenhengia, Anaerobutyricum, Blautia A, Diplocloster, Eisenbergiella, Enterocloster, Lachnoclostridium, Luxibacter, Mediterraneibacter, Muricomes, Robinsonella, Roseburia, Ruminococcus, Scatomonas, Schaedlerella*), *Oscillospirales* (e.g. *Clostridium E, Flavonifractor, Harryflintia, Massiliclostridium*), *Peptostreptococcales* (*Bacilliculturomica, Emergencia, Terrisporobacter*), *Tissierellales* (*Anaerococcus, Peptoniphilus A, Anaerosalibacter*), and *UBA1381* (*Congzhengia*). The vast majority of these species were predicted to transform propionyl-CoA to propionate.

**Figure 6:**
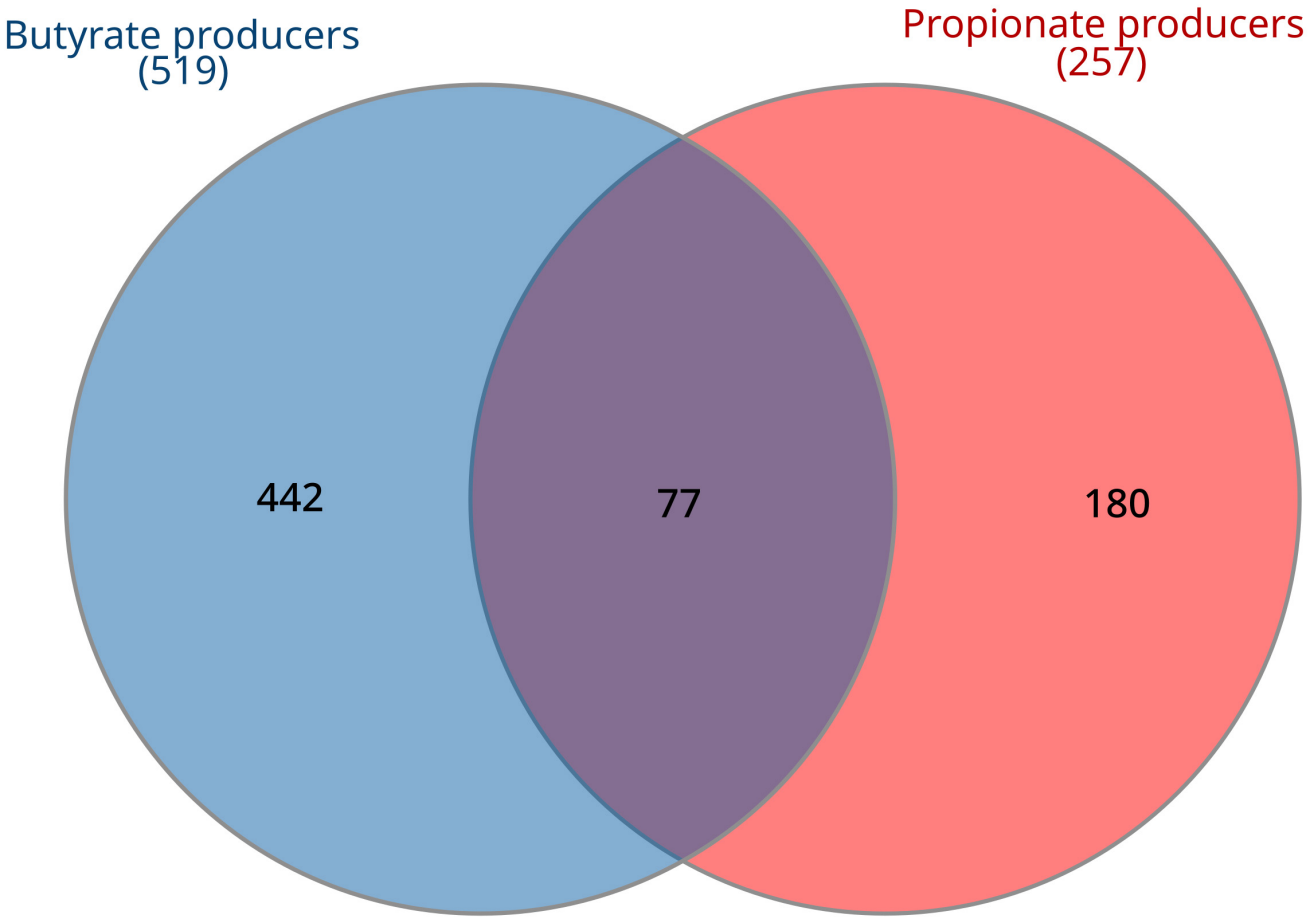
Distribution of predicted complete metabolic pathways in clostridial species (RS + NS) for butyrate and propionate production.

A total of 519 species harbored complete butyrate production pathways, 257 species possessed complete propionate production pathways, and 77 species could produce both SCFAs. 442 butyrate and 180 propionate producers exhibited exclusive production capabilities for only one of these SCFAs (Figure 6).

### 3.6 Profiling of butyrate and propionate producing *Clostridia* in human gut microbiomes

Putative butyrate and propionate producing *Clostridia* were quantified based on the relative abundance of species within the microbiomes.

**Figure 7:**
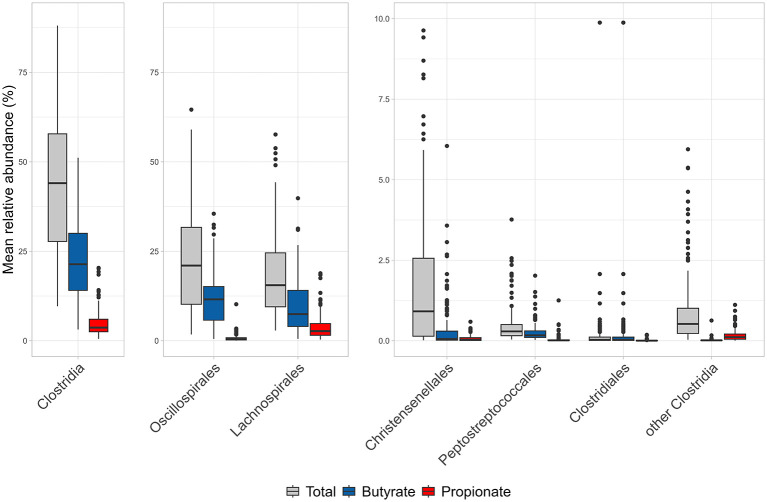
Relative abundance and pathway distribution of butyrate and propionate producers across *Clostridia* taxa. Boxplots showing the mean relative abundance (%) across samples for different taxonomic groups. In each sub-panel, gray boxes represent total abundance, blue boxes indicate butyrate producers, and red boxes indicate propionate producers. Whiskers extend to 1.5 × the interquartile range (IQR) beyond the first and third quartiles.

*Clostridia* potentially producing butyrate accounted on average for 22.2% (median = 21.4%), ranging from 3.2 to 51.1 % (Figure 7). With regard to the routes generating butyrate, the most represented was the acetyl-CoA pathway (median = 21.2%), while glutarate, lysine and succinate semialdehyde pathways were minor (each with a median < 1.5%). The *but* branch was predominant compared to *buk*, in terms of bacterial abundance (median = 19.9 and 7.0%, respectively) (Supplementary Figure 3).

The orders with the highest abundance of putative butyrate producers were *Oscillospirales* and *Lachnospirales*, where species with at least one complete butyrate pathway accounted on average for 11.9% (median = 11.6%) and 9.5% (median = 7.4%), respectively. The abundance of putative butyrate producers within *Christensenellales, Peptostreptococcales, Clostridiales*, and cumulatively the other clostridial orders presented a median relative abundance < 0.2%, much lower compared to that of *Oscillospirales* and *Lachnospirales* (Figure 7).

Of the 50 most abundant *Clostridia* species (Figure 3), 31 were potential butyrate producers, collectively accounting for 12.7% of the microbiome and representing more than half of putative butyrate producers (22.2%). As a whole, a minority of butyrogenic *Clostridia* (76/519) accounted for more than 80% of butyrate producers. The most abundant butyrate producers belonged to the genera *Acetatifactor, Agathobacter, Faecalibacterium, Gemmiger*, and *Roseburia*.

The abundance of putative propionate producer *Clostridia* was lower compared to butyrate producers (Figure 7). Propionigenic *Clostridia* accounted on average for 4.9% (median = 3.7%). Of the two routes that were found complete in *Clostridia*, the propanediol pathway was the most represented (median = 3.0%), while acrylate pathway was the less abundant (median = 0.5%) (Suppl. Figure 3). *Lachnospirales* was the richest order in propionate producers (median = 2.7%), followed by *Oscillospirales* (median = 0.5%). The contribution of *Christensenellales, Peptostreptococcales, Clostridiales*, and cumulatively the other clostridial orders to the propionigenic community was lower, with abundances < 0.12%.

Among the 50 most abundant *Clostridia*, only 6 *Lachnospiraceae* species were putative propionate producers, all harboring the propanediol pathway: *Blautia A faecis, Blautia A. wexlerae, Blautia A massiliensis, Mediterraneibacter faecis, Roseburia inulinivorans*, and *Ruminococcus B gnavus*. Only *R. inulinivorans* was also able to produce butyrate.

RS and NS contributed similarly to the mean abundance of putative butyrate producers, accounting for 12.1 and 10.1%, respectively, whereas putative propionate producers accounted for 3.3 and 1.6% for RS and NS, respectively (Suppl. Spreadsheet 1).

## 4 Discussion

This study aimed to provide a comprehensive overview of all species within the class *Clostridia* identified in the human gut, including both recognized species (RS) and those detected through metagenomic sequencing without formal species assignment (NS). With the aim of creating a valuable reference for future microbiome research and enabling a more accurate interpretation of *Clostridia* diversity within the human gut microbiome, the analysis encompassed phylogenomic relationships, identification of taxonomic inconsistencies, and metabolic reconstruction of SCFA production pathways, complemented by a quantitative profiling of *Clostridia* across 151 healthy human gut microbiomes.

The genomes of all the intestinal *Clostridia* included in the UHGG catalogue were retrieved and reclassified according to the GTDB taxonomy (R214), which assigned 404 species as RS and 1,493 as NS. We adopted the GTDB framework because it relies on a standardized and phylogenomically consistent approach based exclusively on whole-genome sequences and conserved single-copy marker proteins, ensuring objective and reproducible taxonomic assignments. In contrast, the taxonomy based on List of Prokaryotic names with Standing in Nomenclature (LPSN) also incorporates historical and phenotypic criteria. Some discrepancies still exist, likely due to delays in taxon revision and updating. For example, while GTDB recognizes the orders *Lachnospirales* and *Oscillospirales*, these have not yet been validated in LPSN and remain classified under the order *Eubacteriales*.

The orders *Lachnospirales* and *Oscillospirales*, covering the majority of *Clostridia* species, were mostly composed of NS, as already observed (Almeida et al., 2021). In these and the other major clostridial orders, the genomes of NS were smaller than those of RS (Supplementary Figure 4), supporting the hypothesis that uncultivable *Clostridia* may lack functions related to stress defense and/or exhibit more auxotrophies, which makes their isolation challenging, as suggested by (Nayfach et al. 2019).

Genus- and family-level taxonomic assignments are conventionally determined using protein sequence comparisons rather than nucleotide-based metrics (Parks et al., 2018; Zheng et al., 2020). Consistent with this standard framework, AAI and POCP were employed to delineate genera containing at least one RS, applying the widely accepted cutoffs of 65% and 50%, respectively (Konstantinidis et al., 2017; Qin et al., 2014). The use of these established protein-based metrics and validated thresholds minimizes methodological bias and provides an objective basis for assessing existing taxonomic assignments, thereby identifying unresolved issues and inconsistencies in clostridial classification (Riesco and Trujillo, 2024). Of the 77 GTDB genera with multiple species, only 50 met both the minimum AAI and POCP thresholds. Meanwhile, 18 genera failed either AAI or POCP threshold, and 8 failed both (Table 1). Some putative genera could also emerge from merging species with conflicting genus assignments or by refining current genus boundaries. These observations highlight that, despite substantial improvements have already been made in the phylogenomic taxonomy of *Clostridia* (Candeliere et al., 2023; Bello et al., 2024), the delineation of some clostridial genera still needs to be resolved. Otherwise, it should not be excluded that the threshold metrics could be revised to meet the evidence generated from the reconstruction of phylogenomic relationships.

In addition to enabling precise taxonomic and phylogenetic analysis of *Clostridia*, RS genomes and reconstructed MAGs from global-scale genome-resolved metagenomics enabled the prediction of functional capacities in terms of butyrate and propionate production. Butyrate pathways were predicted in 519 of the 1,897 *Clostridia* species (i.e. 27.3%), that were widespread across all major orders, whereas propionate production was much less common compared to butyrate (257/1,897; 13.5%). Examination of the phylogenetic tree revealed that butyrate and propionate production were concentrated within specific clades that did not always coincide with established genera or families. Putative butyrate-producing *Clostridia* were both more diverse (number of species) and more abundant than propionate producers, highlighting their major role within the intestinal ecosystem (Kircher et al., 2022). In terms of abundance within the gut microbiota of 151 healthy subjects, interindividual variability was substantial for the clostridial content, reflecting on the capability of producing butyrate and propionate.

Among the 50 most abundant *Clostridia* species, most were putative butyrate producers. Moreover, 76 out of 519 species accounted for >80% of total butyrate producers, indicating that butyrate production is a function largely driven by a limited number of dominant taxa, i.e. the genera *Acetatifactor, Agathobacter, Faecalibacterium, Gemmiger*, and *Roseburia*, well-known contributors to colonic butyrate pools and often associated with a healthy gut environment (Singh et al., 2023; Zmora et al., 2019; Mann et al., 2024). On the other hand, within the 50 most abundant taxa, only *Blautia A faecis, B. A wexlerae, B. A massiliensis, Mediterraneibacter faecis, Roseburia inulinivorans*, and *Ruminococcus B gnavus* were putative propionate producers, with *R. inulinivorans* also harboring the capacity to produce butyrate. This limited overlap between butyrate and propionate metabolism suggests a clear functional specialization among abundant *Clostridia* species and underscores a marked asymmetry in SCFA metabolism among *Clostridia*, with butyrate production being taxonomically concentrated in this class and functionally dominant, and propionate production distributed across fewer, mostly *Lachnospiraceae*-affiliated, species.

RS and NS similarly contributed to butyrate production within the microbiota herein analyzed, while propionate producers were less evenly distributed. These findings further support the efforts aiming to expand the culturome and to pursue the isolation and characterization of NS. In this context, ER4 sp000765235, the most abundant and prevalent NS, predicted to produce butyrate, was recently isolated and characterized taxonomically as *Hominicoprocola fusiformis* (Afrizal et al., 2022).

The pathways leading to butyrate and propionate production were analyzed in their entirety, encompassing all the clostridial RS and NS, providing for the first time a comprehensive view on the comparative genomics of these pathways in *Clostridia*. The vast majority of putative butyrate producers harbored a complete pathway originating from acetyl-CoA, indicating a direct connection with central carbon metabolism and glycolysis, whereas butyrate production via the glutarate, succinate semialdehyde, and lysine pathways, funneling carbon atoms derived from amino acid metabolism, was much less frequent. More than a hundred species harboring complete *but* and *buk* branches lacked any pathway providing them with crotonyl-CoA. It should be taken into account that gaps in genome sequencing and assembly, that especially affect NS, may have led to an underestimation of pathway completeness. Another possible cause of the apparent underrepresentation of complete pathways may lie in the GapSeq tool, which prioritizes UniProt-reviewed proteins as queries for homology searches. These reference proteins may originate from bacteria phylogenetically distant from the *Clostridia* class and, consequently, may be less effective in detecting clostridial homologs, if present. For instance, it is unlikely that the glutarate pathway, comprising four metabolic steps, would be present in 78 species, all of them lacking only 2-oxoglutarate reductase. This indicates that the enzyme is not being detected or discarded possibly due to limitations in the query sequence.

Propionate producers were concentrated within specific clades, not necessarily coinciding with homogeneous taxonomic entities (except for *Blautia A, Eisenbergiella, Clostridium E*, and a few other genera). In agreement with previous studies, the propanediol pathway was the most common, followed by the acrylate one, while the succinate pathway, utilized by *Bacteroides*, the main intestinal propionate producers, was never found to be complete (Reichardt et al., 2014). Butyrate and propionate pathways coexisted in a minority of the species (77). Among them, some species of *Lachnospiraceae* (*C. catus* and *R. inulinivorans*) switch from butyrate to propionate production on different substrates (Reichardt et al., 2014).

Despite the lack of acrylate and propanediol pathways, the widespread occurrence of phosphate propionyltransferase and propionate kinase suggests that these enzymes might function in additional pathways, such as the degradation of several amino acids that yield 2-oxobutanoate and then propionyl-CoA and/or propionyl-phosphate. This observation deserves further investigation.

Enzymes involved in acetate and lactate metabolism were identified in numerous species (1,465 and 1,138, respectively), showing substantial overlap with each other and with those predicted to convert acetyl-CoA into butyrate. With regard to the utilization of acetate and lactate in butyrate production, an important cross-feeding occurring in the intestine, due to the reversible nature of many of these reactions (the direction depending on the energy and the redox status of the cell), it remains to be clarified whether such a great number of species is actually capable of transforming acetate and/or lactate into butyrate or whether acetate, lactate and butyrate are just alternative fermentation end products, toward which the carbon flow diverges at the level of pyruvate.

The widespread presence of both butyrate and propionate pathways across phylogenetically distant orders, as well as their relatively homogeneous occurrence within specific clades, suggests that it is worth considering how these fermentation pathways evolved and were inherited throughout the evolutionary history of the class, ultimately giving rise to their present polyphyletic distribution.

The outcomes of this study can help the scientific community better understand *Clostridia*, key players in all anoxic ecosystems, including the gut, by deepening awareness of their vast diversity and providing new insights into their phylogenetic relationships. Moreover, the knowledge of taxa involved in butyrate and propionate biosynthesis is expanded, highlighting the role of yet uncharacterized species.

## Data Availability

The datasets presented in this study can be found in online repositories. The names of the repository/repositories and accession number(s) can be found in the article/Supplementary material.
