# Supplementary material for: A genomic atlas of gut clostridia: phylogeny, butyrate, and propionate production
Source: Front Microbiol. 2026 Apr 10;17:1761627. doi: 10.3389/fmicb.2026.1761627 (PMC13106387; doi:10.3389/fmicb.2026.1761627)
Supplement: Supplementary file 1 [file Supplementary_File_1.docx]

Supplementary Material

**A Genomic Atlas of Gut Clostridia: Phylogeny, Butyrate, and Propionate Production**

**Laura Sola^1^**^†^**, Francesco Candeliere^1^**^†^**, Enrico Busi^1^, Stefano Raimondi^1,2^, Alberto Amaretti^1,2^, Maddalena Rossi^1,2*^**

*^1^* *Department of Life Sciences, University of Modena and Reggio Emilia, Modena, 41125, Italy.*

*^2^ Biogest-Siteia, University of Modena and Reggio Emilia, Modena, 41125 Italy.*

†These authors have contributed equally to this work

*** Correspondence:**Maddalena Rossi
maddalena.rossi@unimore.it

**Supplementary Table 1.** Accession numbers and bioproject code of the 151 metagenomes utilized in this work. The number and length of reads are reported. First three letters in sample’s name indicate geographic origin: China (CHN), Ethiopia (ETH), Germany (GER), Ghana (GHA), Italy (ITA), Japan (JAP), Madagascar (MAD), Mongolia (MON), Peru (PER), Sweden (SWE), Tanzania (TAN), United States of America (USA).

| **Sample** | **Accession number** | **Bioproject** | **N° reads** | **Reads length** |
| --- | --- | --- | --- | --- |
| CHN-01 | SRR6474215 | PRJNA375935 | 7.22E+09 | 150 |
| CHN-02 | SRR6474229 | PRJNA375935 | 3.16E+09 | 150 |
| CHN-03 | SRR6474231 | PRJNA375935 | 5.93E+09 | 150 |
| CHN-04 | SRR6474260 | PRJNA375935 | 2.17E+09 | 150 |
| CHN-05 | SRR6474261 | PRJNA375935 | 4.18E+09 | 150 |
| CHN-06 | SRR6474266 | PRJNA375935 | 3.63E+09 | 150 |
| CHN-07 | SRR6474278 | PRJNA375935 | 3.64E+09 | 150 |
| CHN-08 | SRR6474283 | PRJNA375935 | 3.39E+09 | 150 |
| CHN-09 | SRR6474290 | PRJNA375935 | 2.61E+09 | 150 |
| CHN-10 | SRR6474291 | PRJNA375935 | 3.36E+09 | 150 |
| CHN-11 | SRR10680552 | PRJNA557323 | 2.55E+07 | 150 |
| CHN-12 | SRR10680551 | PRJNA557323 | 2.59E+07 | 150 |
| CHN-13 | SRR10680550 | PRJNA557323 | 2.47E+07 | 150 |
| CHN-14 | SRR10680549 | PRJNA557323 | 2.74E+07 | 150 |
| CHN-15 | SRR10680548 | PRJNA557323 | 2.66E+07 | 150 |
| CHN-16 | SRR10680547 | PRJNA557323 | 3.03E+07 | 150 |
| CHN-17 | SRR10680545 | PRJNA557323 | 2.90E+07 | 150 |
| CHN-18 | SRR10680544 | PRJNA557323 | 2.51E+07 | 150 |
| CHN-19 | SRR10680543 | PRJNA557323 | 2.93E+07 | 150 |
| CHN-20 | SRR10680542 | PRJNA557323 | 2.76E+07 | 150 |
| CHN-21 | SRR10680541 | PRJNA557323 | 2.73E+07 | 150 |
| CHN-22 | SRR10680540 | PRJNA557323 | 2.49E+07 | 150 |
| CHN-23 | SRR10680445 | PRJNA557323 | 2.50E+07 | 150 |
| CHN-24 | SRR10680443 | PRJNA557323 | 2.73E+07 | 150 |
| CHN-25 | SRR10680442 | PRJNA557323 | 2.73E+07 | 150 |
| CHN-26 | SRR10680441 | PRJNA557323 | 2.49E+07 | 150 |
| CHN-27 | SRR10680439 | PRJNA557323 | 2.50E+07 | 150 |
| ETH-01 | SRR8784372 | PRJNA504891 | 3.43E+07 | 100 |
| ETH-02 | SRR8784387 | PRJNA504891 | 1.94E+07 | 100 |
| ETH-03 | SRR8784374 | PRJNA504891 | 2.35E+07 | 100 |
| ETH-04 | SRR8784376 | PRJNA504891 | 2.99E+07 | 100 |
| ETH-05 | SRR8784385 | PRJNA504891 | 3.02E+07 | 100 |
| ETH-06 | SRR8784383 | PRJNA504891 | 4.57E+07 | 100 |
| ETH-07 | SRR8784379 | PRJNA504891 | 2.50E+07 | 100 |
| ETH-08 | SRR8784390 | PRJNA504891 | 2.50E+07 | 100 |
| ETH-09 | SRR8784391 | PRJNA504891 | 2.28E+07 | 100 |
| ETH-10 | SRR8784395 | PRJNA504891 | 2.42E+07 | 100 |
| ETH-11 | SRR8784394 | PRJNA504891 | 1.72E+07 | 100 |
| GER-01 | ERR1912947 | PRJEB17784 | 2.35E+09 | 150 |
| GER-02 | ERR1912948 | PRJEB17784 | 1.99E+09 | 150 |
| GER-03 | ERR1912962 | PRJEB17784 | 1.95E+09 | 150 |
| GER-04 | ERR1912963 | PRJEB17784 | 1.63E+09 | 150 |
| GER-05 | ERR1912992 | PRJEB17784 | 1.31E+09 | 150 |
| GER-06 | ERR1912993 | PRJEB17784 | 1.10E+09 | 150 |
| GER-07 | ERR1913030 | PRJEB17784 | 2.80E+09 | 150 |
| GER-08 | ERR1913031 | PRJEB17784 | 2.38E+09 | 150 |
| GER-09 | ERR1913117 | PRJEB17784 | 2.21E+09 | 150 |
| GER-10 | ERR1913118 | PRJEB17784 | 1.87E+09 | 150 |
| GHA-01 | SRR8791364 | PRJNA529124 | 4.99E+09 | 150 |
| GHA-02 | SRR8791365 | PRJNA529124 | 3.98E+09 | 150 |
| GHA-03 | SRR8791366 | PRJNA529124 | 5.38E+09 | 150 |
| GHA-04 | SRR8791367 | PRJNA529124 | 4.02E+09 | 150 |
| GHA-05 | SRR8791368 | PRJNA529124 | 5.66E+09 | 150 |
| GHA-06 | SRR8791369 | PRJNA529124 | 2.40E+09 | 150 |
| GHA-07 | SRR8791370 | PRJNA529124 | 6.73E+09 | 150 |
| GHA-08 | SRR8791371 | PRJNA529124 | 4.58E+09 | 150 |
| GHA-09 | SRR8791372 | PRJNA529124 | 5.02E+09 | 150 |
| GHA-10 | SRR8791373 | PRJNA529124 | 4.47E+09 | 150 |
| ITA-01 | SRR1930248 | PRJNA278393 | 8.18E+08 | 150 |
| ITA-02 | SRR1930250 | PRJNA278393 | 3.17E+08 | 150 |
| ITA-03 | SRR1930251 | PRJNA278393 | 4.50E+08 | 150 |
| ITA-04 | SRR1930253 | PRJNA278393 | 1.90E+09 | 150 |
| ITA-05 | SRR1930255 | PRJNA278393 | 1.40E+09 | 150 |
| ITA-06 | SRR1930777 | PRJNA278393 | 5.36E+08 | 150 |
| ITA-07 | SRR1931170 | PRJNA278393 | 3.21E+09 | 150 |
| ITA-08 | SRR1931173 | PRJNA278393 | 4.63E+09 | 150 |
| ITA-09 | SRR1931177 | PRJNA278393 | 5.48E+08 | 150 |
| ITA-10 | SRR1931178 | PRJNA278393 | 6.44E+08 | 150 |
| JAP-01 | DRR127532 | PRJDB4176 | 5.15E+09 | 150 |
| JAP-02 | DRR127537 | PRJDB4176 | 5.93E+09 | 150 |
| JAP-03 | DRR127552 | PRJDB4176 | 4.02E+09 | 150 |
| JAP-04 | DRR127597 | PRJDB4176 | 5.02E+09 | 150 |
| JAP-05 | DRR127619 | PRJDB4176 | 5.12E+09 | 150 |
| JAP-06 | DRR127634 | PRJDB4176 | 1.04E+10 | 150 |
| JAP-07 | DRR127649 | PRJDB4176 | 5.94E+09 | 150 |
| JAP-08 | DRR127672 | PRJDB4176 | 6.20E+09 | 150 |
| JAP-09 | DRR127683 | PRJDB4176 | 6.29E+09 | 150 |
| JAP-10 | DRR127713 | PRJDB4176 | 7.17E+09 | 150 |
| MAD-01 | SRR7658639 | PRJNA485056 | 1.30E+08 | 150 |
| MAD-02 | SRR7658640 | PRJNA485056 | 1.55E+08 | 150 |
| MAD-03 | SRR7658641 | PRJNA485056 | 1.16E+08 | 150 |
| MAD-04 | SRR7658643 | PRJNA485056 | 1.12E+08 | 150 |
| MAD-05 | SRR7658644 | PRJNA485056 | 1.14E+08 | 150 |
| MAD-06 | SRR7658646 | PRJNA485056 | 1.37E+08 | 150 |
| MAD-07 | SRR7658647 | PRJNA485056 | 1.24E+08 | 150 |
| MAD-08 | SRR7658648 | PRJNA485056 | 1.29E+08 | 150 |
| MAD-09 | SRR7658665 | PRJNA485056 | 8.73E+09 | 150 |
| MAD-10 | SRR7658668 | PRJNA485056 | 1.80E+08 | 150 |
| MON-01 | SRR3992957 | PRJNA328899 | 5.22E+09 | 150 |
| MON-02 | SRR3992972 | PRJNA328899 | 6.40E+09 | 150 |
| MON-03 | SRR3992974 | PRJNA328899 | 5.77E+09 | 150 |
| MON-04 | SRR3992975 | PRJNA328899 | 5.59E+09 | 150 |
| MON-05 | SRR3992977 | PRJNA328899 | 5.44E+09 | 150 |
| MON-06 | SRR3992979 | PRJNA328899 | 6.38E+09 | 150 |
| MON-07 | SRR3992982 | PRJNA328899 | 5.32E+09 | 150 |
| MON-08 | SRR3992983 | PRJNA328899 | 5.94E+09 | 150 |
| MON-09 | SRR3992989 | PRJNA328899 | 6.03E+09 | 150 |
| MON-10 | SRR3993010 | PRJNA328899 | 5.68E+09 | 150 |
| PER-01 | SRR1761667 | PRJNA268964 | 1.29E+09 | 150 |
| PER-02 | SRR1761668 | PRJNA268964 | 1.18E+09 | 150 |
| PER-03 | SRR1761670 | PRJNA268964 | 1.52E+09 | 150 |
| PER-04 | SRR1761672 | PRJNA268964 | 1.44E+09 | 150 |
| PER-05 | SRR1761675 | PRJNA268964 | 1.57E+09 | 150 |
| PER-06 | SRR1761698 | PRJNA268964 | 5.03E+09 | 150 |
| PER-07 | SRR1761709 | PRJNA268964 | 4.31E+09 | 150 |
| PER-08 | SRR1761711 | PRJNA268964 | 4.27E+09 | 150 |
| PER-09 | SRR1761716 | PRJNA268964 | 4.75E+09 | 150 |
| PER-10 | SRR1761717 | PRJNA268964 | 4.45E+09 | 150 |
| SWE-01 | ERS554193 | PRJEB7369 | 1.27E+08 | 100 |
| SWE-02 | ERR636369 | PRJEB7369 | 9.12E+07 | 100 |
| SWE-03 | ERR636383 | PRJEB7369 | 7.10E+07 | 100 |
| SWE-04 | ERR636385 | PRJEB7369 | 1.03E+08 | 100 |
| SWE-05 | ERR636375 | PRJEB7369 | 1.78E+08 | 100 |
| SWE-06 | ERR636405 | PRJEB7369 | 8.48E+07 | 100 |
| SWE-07 | ERR636371 | PRJEB7369 | 9.41E+07 | 100 |
| SWE-08 | ERR636411 | PRJEB7369 | 8.67E+07 | 100 |
| SWE-09 | ERR636351 | PRJEB7369 | 5.67E+07 | 100 |
| SWE-10 | ERR636373 | PRJEB7369 | 7.78E+07 | 100 |
| SWE-11 | ERR636353 | PRJEB7369 | 1.12E+08 | 100 |
| SWE-12 | ERR636391 | PRJEB7369 | 6.57E+07 | 100 |
| SWE-13 | ERR636389 | PRJEB7369 | 9.99E+07 | 100 |
| SWE-14 | ERR636355 | PRJEB7369 | 1.01E+08 | 100 |
| SWE-15 | ERR636359 | PRJEB7369 | 8.42E+07 | 100 |
| SWE-16 | ERR636363 | PRJEB7369 | 9.76E+07 | 100 |
| TAN-01 | SRR1929408 | PRJNA278393 | 6.14E+09 | 150 |
| TAN-02 | SRR1929563 | PRJNA278393 | 2.66E+09 | 150 |
| TAN-03 | SRR1929574 | PRJNA278393 | 1.98E+09 | 150 |
| TAN-04 | SRR1930122 | PRJNA278393 | 2.93E+09 | 150 |
| TAN-05 | SRR1930136 | PRJNA278393 | 2.30E+09 | 150 |
| TAN-06 | SRR1930138 | PRJNA278393 | 9.24E+08 | 150 |
| TAN-07 | SRR1930144 | PRJNA278393 | 9.41E+08 | 150 |
| TAN-08 | SRR1930179 | PRJNA278393 | 9.81E+08 | 150 |
| TAN-09 | SRR1930187 | PRJNA278393 | 7.60E+08 | 150 |
| TAN-10 | SRR1930244 | PRJNA278393 | 1.53E+09 | 150 |
| USA-01 | SRR1761676 | PRJNA268964 | 3.77E+09 | 150 |
| USA-02 | SRR1761682 | PRJNA268964 | 3.98E+09 | 150 |
| USA-03 | SRR1761683 | PRJNA268964 | 3.47E+09 | 150 |
| USA-04 | SRR1761684 | PRJNA268964 | 4.41E+09 | 150 |
| USA-05 | SRR1761686 | PRJNA268964 | 4.55E+09 | 150 |
| USA-06 | SRR1761687 | PRJNA268964 | 4.16E+09 | 150 |
| USA-07 | SRR1761689 | PRJNA268964 | 3.71E+09 | 150 |
| USA-08 | SRR1761690 | PRJNA268964 | 5.21E+09 | 150 |
| USA-09 | SRR1761694 | PRJNA268964 | 4.24E+09 | 150 |
| USA-10 | SRR1761697 | PRJNA268964 | 4.87E+09 | 150 |
| USA-11 | ERR2641799 | PRJEB27308 | 1.37E+07 | 150 |
| USA-12 | ERR2641792 | PRJEB27308 | 9.17E+06 | 150 |
| USA-13 | ERR2641793 | PRJEB27308 | 9.40E+06 | 150 |
| USA-14 | ERR2641795 | PRJEB27308 | 1.22E+07 | 150 |
| USA-15 | ERR2641798 | PRJEB27308 | 1.92E+07 | 150 |
| USA-16 | ERR2641800 | PRJEB27308 | 1.11E+07 | 150 |
| USA-17 | ERR2641801 | PRJEB27308 | 1.04E+07 | 150 |

**Supplementary Table 2.** Number of species in the orders of class *Clostridia*, subdivided as NS and RS.

| Order | tot |  | NS | |  | RS |  |
| --- | --- | --- | --- | --- | --- | --- | --- |
|  |  |  | tot | (%) |  | tot |  |
| *Christensenellales* | 248 |  | 213 | (85.9) |  | 35 |  |
| *Clostridiales* | 47 |  | 16 | (34.0) |  | 31 |  |
| *Eubacteriales* | 6 |  | 2 | (33.3) |  | 4 |  |
| *HGM11327* | 3 |  | 3 | (100) |  | 0 |  |
| *HGM11514* | 3 |  | 3 | (100) |  | 0 |  |
| *Lachnospirales* | 512 |  | 368 | (71.9) |  | 144 |  |
| *Monoglobales* | 13 |  | 11 | (84.6) |  | 2 |  |
| *Oscillospirales* | 755 |  | 645 | (85.4) |  | 110 |  |
| *Peptostreptococcales* | 76 |  | 47 | (61.8) |  | 29 |  |
| *RGIG6154* | 1 |  | 1 | (100) |  | 0 |  |
| *RUG12999* | 1 |  | 1 | (100) |  | 0 |  |
| *Saccharofermentanales* | 3 |  | 3 | (100) |  | 0 |  |
| *TANB77* | 121 |  | 119 | (98.3) |  | 2 |  |
| *Tissierellales* | 70 |  | 25 | (35.7) |  | 45 |  |
| *UBA1212* | 6 |  | 5 | (83.3) |  | 1 |  |
| *UBA1381* | 18 |  | 17 | (94.4) |  | 1 |  |
| *UMGS1810* | 7 |  | 7 | (100) |  | 0 |  |
| *UMGS1840* | 2 |  | 2 | (100) |  | 0 |  |
| *UMGS1883* | 5 |  | 5 | (100) |  | 0 |  |

**Supplementary Table 3:** Groups of at least two species with both AAI and POCP above thresholds for genus delineation that could be obtained by merging (*) or restricting (#) nominal genera. When applicable, the belonging to groups defined in Table 1 is reported or defined by 0 when the species is a singleton.

|  |  |  | minAAI  % | minPOCP  % |
| --- | --- | --- | --- | --- |
| 0, 0 | *Anaerosalibacter massiliensis* and *Sporanaerobacter acetigenes* | * | 68.4 | 54.9 |
| 0, A | *Intestinibacter bartlettii, Terrisporobacter petrolearius*, *Terrisporobacter othiniensis* | * | 66.0 | 53.9 |
| 0, A | *Romboutsia timonensis, Paraclostridium tenue, Paraclostridium sordellii, Paraclostridium dentum* | * | 66.2 | 52.8 |
| 0, A | *Levyella massiliensis*, *Murdochiella massiliensis*, *Murdochiella vaginalis* | * | 75.7 | 69.6 |
| B | *Acutalibacter timonensis*, *Acutalibacter stercorigallinarum*, *Acutalibacter pullicola*, *Acutalibacter stercoravium* | # | 68.8 | 53.7 |
| B | *Agathobaculum butyriciproducens*, *Agathobaculum desmolans*, *Agathobaculum pullistercoris*, *Agathobaculum stercoravium* | # | 75.2 | 53.4 |
| B | *Anaerotruncus rubiinfantis*, *Anaerotruncus massiliensis* | # | 67.0 | 51.2 |
| B | *Blautia argi*, *Blautia hansenii*, *Blautia pullicola*, *Blautia ornithocaccae* | # | 70.2 | 50.2 |
| B | *Blautia avicola, Blautia merdigallinarum* | # | 87.2 | 65.3 |
| B | *Blautia hominis, Blautia coccoides* | # | 87.9 | 73.3 |
| B | *Clostridium Q saccharolyticum A*, *Clostridium Q fessum* |  | 66.0 | 52.0 |
| B | *Dysosmobacter faecalis*, *Dysosmobacter welbionis*, *Dysosmobacter avistercoris* |  | 70.1 | 50.1 |
| B | *Eisenbergiella porci*, *Eisenbergiella tayi* | # | 78.3 | 56.4 |
| B | *Eisenbergiella stercoravium*, *Eisenbergiella intestinigallinarum* | # | 90.7 | 72.6 |
| B | *Fournierella massiliensis*, *Fournierella excrementavium* |  | 67.9 | 52.1 |
| B | *Limiplasma stercoravium*, *Limiplasma merdipullorum* |  | 70.3 | 60.4 |
| B | *Mediterraneibacter massiliensis*, *Mediterraneibacter lactaris*, *Mediterraneibacter faecigallinarum*, *Mediterraneibacter caccogallinarum*, *Mediterraneibacter norwichensis*, *Mediterraneibacter torques*, *Mediterraneibacter vanvlietii*, *Mediterraneibacter intestinigallinarum* | # | 68.8 | 50.5 |
| C | *Anaerococcus hydrogenalis*, *Anaerococcus senegalensis*, *Anaerococcus obesiensis*, *Anaerococcus vaginalis B*, *Anaerococcus rubeinfantis*, *Anaerococcus jeddahensis* | # | 84.1 | 61.4 |
| C | *Anaerococcus nagyae*, *Anaerococcus octavius*, *Anaerococcus vaginimassiliensis* | # | 66.1 | 61.1 |
| C | *Anaerococcus prevotii*, *Anaerococcus lactolyticus*, *Anaerococcus degeneri*, *Anaerococcus murdochii* | # | 67.1 | 61.0 |
| C | *Butyribacter intestini*, *Butyribacter hominis* | # | 65.2 | 52.2 |
| C | *Eubacterium R faecigallinarum*, *Eubacterium R faecavium*, *Eubacterium R faecipullorum*, *Eubacterium R faecale* | # | 65.5 | 61.1 |
| C | *Peptoniphilus A lacydonensis*, *Peptoniphilus A senegalensis*, *Peptoniphilus A gorbachii*, *Peptoniphilus A grossensis*, *Peptoniphilus A phoceensis*, *Peptoniphilus A harei A*, *Peptoniphilus A harei* | # | 79.8 | 66.1 |
| D | *Blautia A massiliensis*, *Blautia A schinkii*, *Blautia A caecimuris*, *Blautia A obeum*, *Blautia A intestinalis*, *Blautia A faecis*, *Blautia A luti*, *Blautia A wexlerae B*, *Blautia A wexlerae* | # | 67.8 | 50.8 |
| D | *Clostridium disporicum B, Clostridium cuniculi, Clostridium saudiense Clostridium celatum* | # | 69.3 | 56.2 |
| D | *Clostridium J ihumii*, *Clostridium J senegalense* | # | 69.5 | 58.6 |
| D | *Clostridium neonatale, Clostridium butyricum*, and *Clostridium beijerinckii* | # | 67.5 | 51.7 |
| D | *Clostridium septicum, Clostridium nigeriense, Clostridium tertium* | # | 68.4 | 56.1 |
| D | *Enterocloster asparagiformis*, *Enterocloster lavalensis* | # | 95.1 | 79.8 |
| D | *Enterocloster citroniae*, *Enterocloster aldenensis*, *Enterocloster pacaense*, *Enterocloster bolteae*, *Enterocloster clostridioformis*, *Enterocloster clostridioformis A* | # | 74.6 | 50.9 |
| D | *Enterocloster faecavium*, *Enterocloster excrementigallinarum* | # | 87.7 | 64.7 |
| D | *Sarcina massiliamazoniense*, *Sarcina mediterraneense* | # | 65.9 | 56.4 |


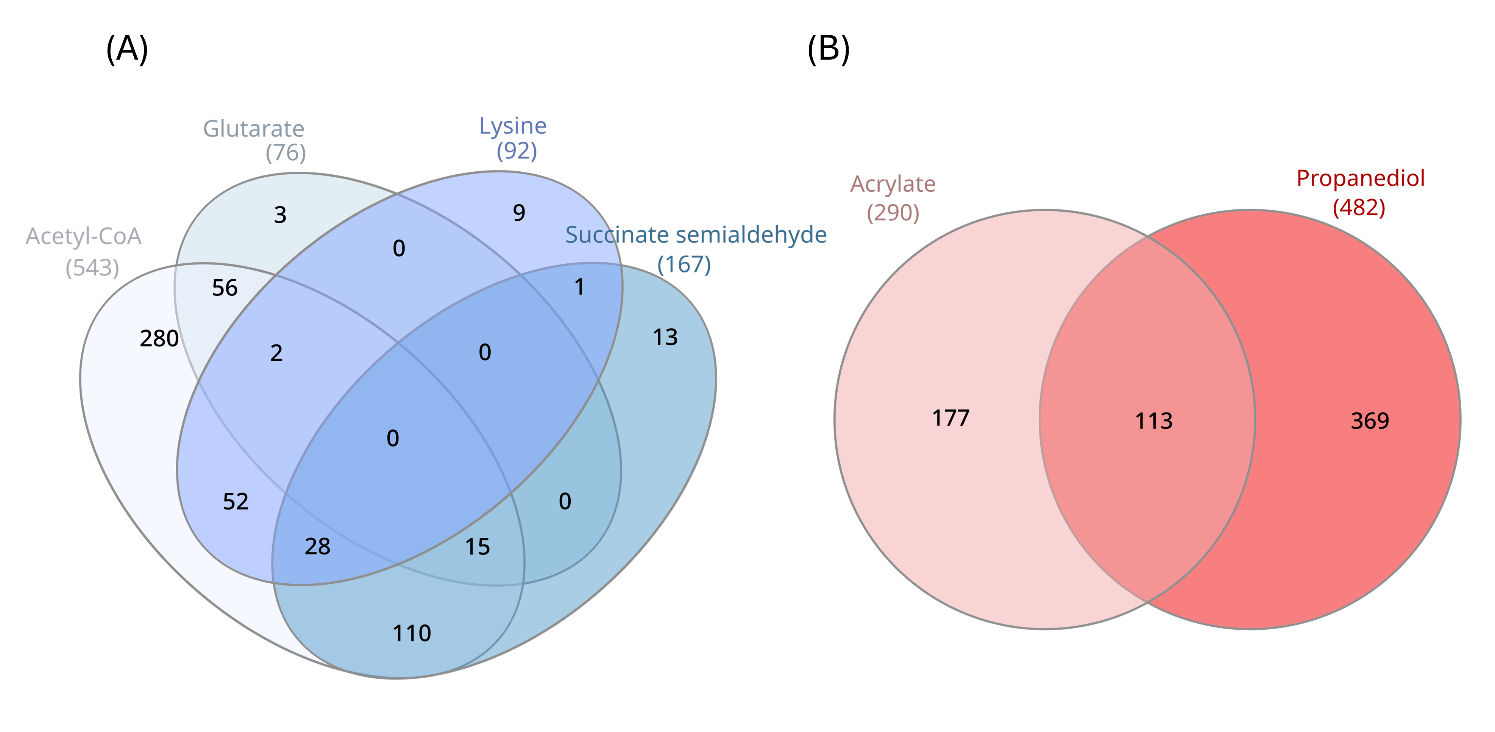


**Supplementary Figure 2.** Distribution of clostridial species encoding butyrate (A) and propionate (B) metabolic pathways, allowing one missing reaction.


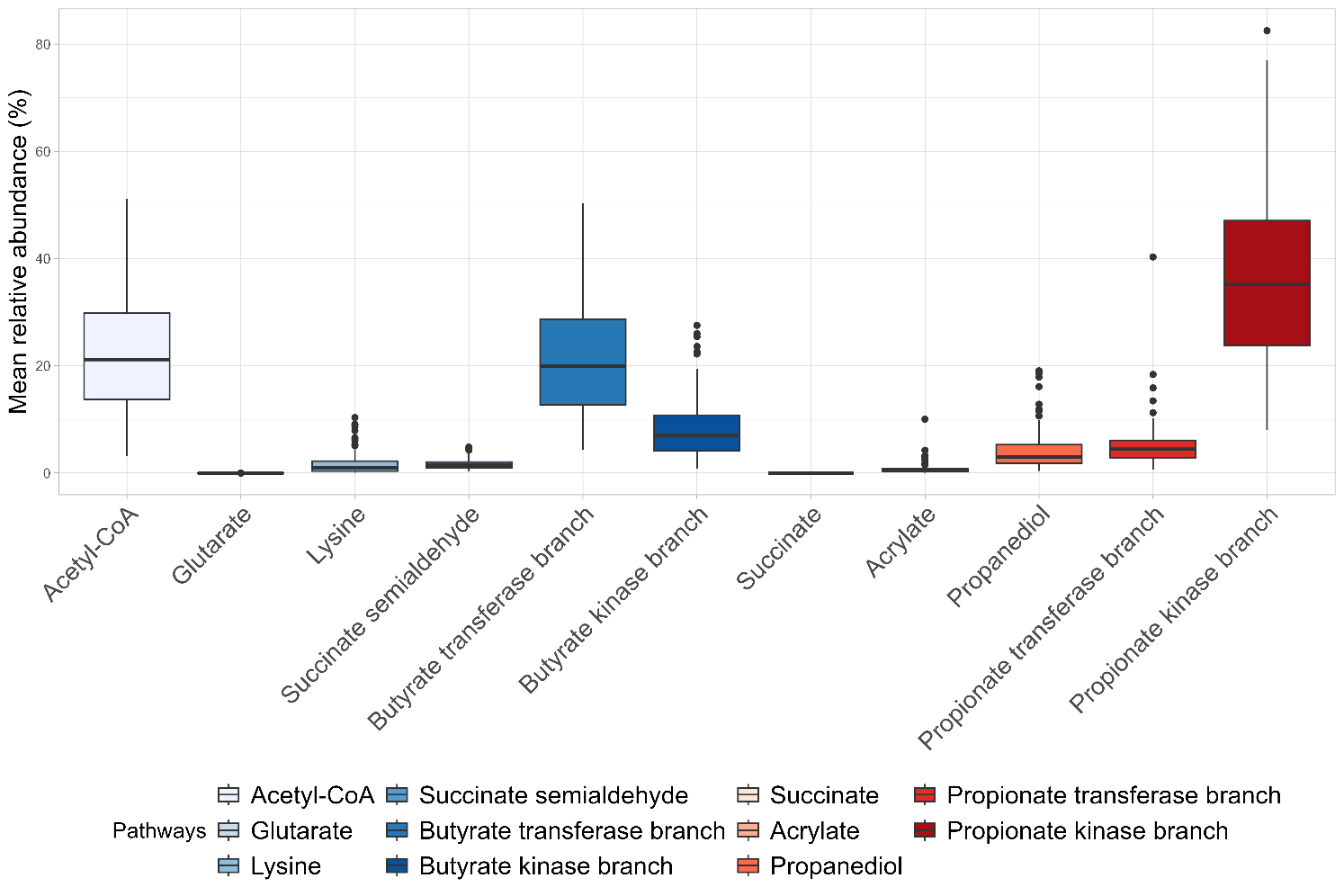


**Supplementary Figure 3.** Relative abundance (%) of species carrying specific complete biosynthetic pathways. Butyrate pathways are shown in blue gradient: acetyl-CoA, glutarate, lysine, and succinate semialdehyde pathways, along with the butyrate CoA-transferase and butyrate kinase terminal branches. Propionate pathways are shown in red gradient: succinate, acrylate and propanediol pathways, along with the propionate CoA-transferase and propionate kinase terminal branches. Whiskers extend to 1.5 × the interquartile range (IQR) beyond the first and third quartiles.


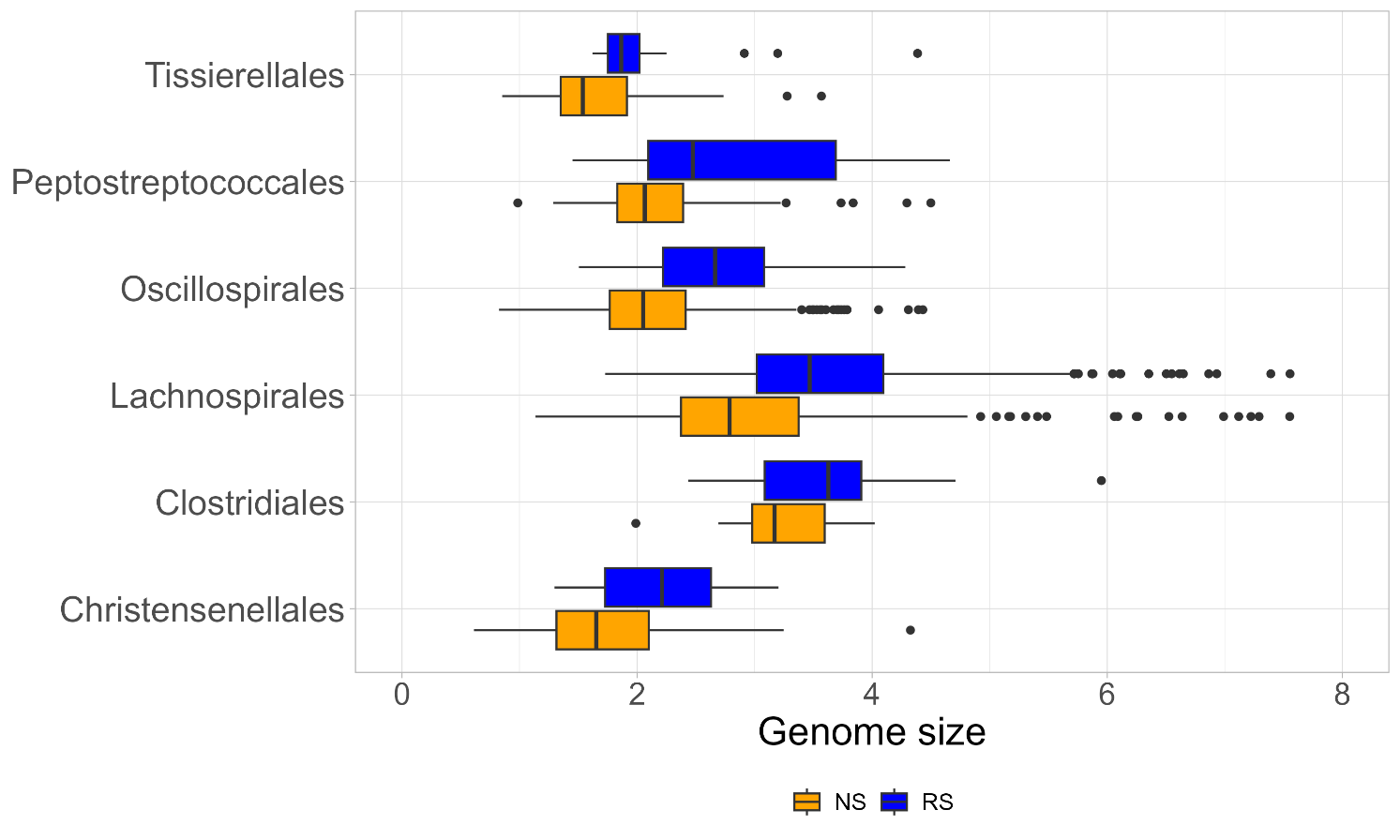


**Supplementary Figure 4.** Boxplot of genome size of RS and NS ascribed to most important orders. Whiskers extend to 1.5 × the interquartile range (IQR) beyond the first and third quartiles.
