# Supplementary material for: A genomic atlas of gut clostridia: phylogeny, butyrate, and propionate production
Source: Front Microbiol. 2026 Apr 10;17:1761627. doi: 10.3389/fmicb.2026.1761627 (PMC13106387; doi:10.3389/fmicb.2026.1761627)

Supplementary Figure 1: Phylogenetic tree of the 1897 species belonging to class *Clostridia* identified in human gut microbiota. The tree is rooted on *Bacillus licheniformis*. Leaves marked with * represent RS.

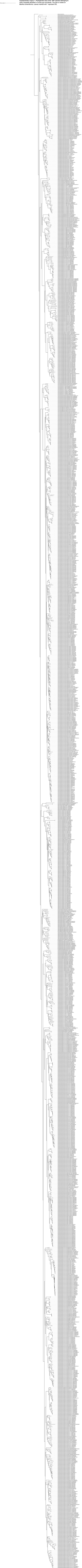

Supplement: Supplementary file 5 [file Image_1.pdf]
